# Context-specific network modeling identifies new crosstalk in β-adrenergic cardiac hypertrophy

**DOI:** 10.1371/journal.pcbi.1008490

**Published:** 2020-12-18

**Authors:** Ali Khalilimeybodi, Alexander M. Paap, Steven L. M. Christiansen, Jeffrey J. Saucerman

**Affiliations:** 1 Department of Biomedical Engineering, University of Virginia, Charlottesville, Virginia, United States of America; 2 Robert M. Berne Cardiovascular Research Center, University of Virginia, Charlottesville, Virginia, United States of America; National Institutes of Health, UNITED STATES

## Abstract

Cardiac hypertrophy is a context-dependent phenomenon wherein a myriad of biochemical and biomechanical factors regulate myocardial growth through a complex large-scale signaling network. Although numerous studies have investigated hypertrophic signaling pathways, less is known about hypertrophy signaling as a whole network and how this network acts in a context-dependent manner. Here, we developed a systematic approach, CLASSED (Context-specific Logic-bASed Signaling nEtwork Development), to revise a large-scale signaling model based on context-specific data and identify main reactions and new crosstalks regulating context-specific response. CLASSED involves four sequential stages with an automated validation module as a core which builds a logic-based ODE model from the interaction graph and outputs the model validation percent. The context-specific model is developed by estimation of default parameters, classified qualitative validation, hybrid Morris-Sobol global sensitivity analysis, and discovery of missing context-dependent crosstalks. Applying this pipeline to our prior-knowledge hypertrophy network with context-specific data revealed key signaling reactions which distinctly regulate cell response to isoproterenol, phenylephrine, angiotensin II and stretch. Furthermore, with CLASSED we developed a context-specific model of β-adrenergic cardiac hypertrophy. The model predicted new crosstalks between calcium/calmodulin-dependent pathways and upstream signaling of Ras in the ISO-specific context. Experiments in cardiomyocytes validated the model’s predictions on the role of CaMKII-Gβγ and CaN-Gβγ interactions in mediating hypertrophic signals in ISO-specific context and revealed a difference in the phosphorylation magnitude and translocation of ERK1/2 between cardiac myocytes and fibroblasts. CLASSED is a systematic approach for developing context-specific large-scale signaling networks, yielding insights into new-found crosstalks in β-adrenergic cardiac hypertrophy.

## Author summary

Pathological cardiac hypertrophy is a disease in which the heart grows abnormally in response to different motivators such as high blood pressure or variations in hormones and growth factors. The shape of the heart after its growth depends on the context in which it grows. Since cell signaling in the cardiac cells plays a key role in the determination of heart shape, a thorough understanding of cardiac cells signaling in each context enlightens the mechanisms which control response of cardiac cells. However, cell signaling in cardiac hypertrophy comprises a complex web of pathways with numerous interactions, and predicting how these interactions control the hypertrophic signal in each context is not achievable by only experiments or general computational models. To address this need, we developed an approach to bring together the experimental data of each context with a signaling network curated from literature to identify the main players of cardiac cells response in each context and attain the context-specific models of cardiac hypertrophy. By utilizing our approach, we identified the main regulators of cardiac hypertrophy in four important contexts. We developed a network model of β-adrenergic cardiac hypertrophy, and predicted and validated new interactions that regulate cardiac cells response in this context.

## Introduction

Cardiac hypertrophy, a putative risk factor of heart failure, is a context-dependent phenomenon [1–3] which is regulated by a broad range of biochemical and biomechanical factors interacting in a complex network of signaling pathways [4,5]. Homogenous growth of the heart occurs in the contexts of exercise and pregnancy [6,7]. But pathological contexts such as hypertension lead to an irreversible thickening of the heart wall [8–10], distinct from heart chamber dilation which is triggered in the contexts of myocardium infarction and valvular heart disease [11]. Identifying signaling pathways specific to each context has long been of interest [12], but most experimental studies have focused on single stimuli and isolated pathways for practicality and limited resources [13–15]. While computational models of cardiac hypertrophy signaling [16–21] address this issue to a certain extent by considering multiple pathways, predicting interactions between pathways in each context has remained a challenge due to the size and connectivity of cardiac signaling network.

Recent advances in experimental and computational approaches [22–27] facilitate the investigation of whole signaling networks and inference of new causal interactions. Among the established approaches to derive a network model [28–30], data-driven approaches that do not use prior knowledge has been widely applied for modeling gene regulatory and metabolic networks [31,32]. But modeling large and complex signaling networks via this method is not feasible owing to its low performance in model scale-up and interpretability [33]. In contrast, prior knowledge approaches have been implemented successfully by several groups to model large-scale signaling networks [34,35]. Yet prior knowledge models comprise numerous parameters to train, and only limited experimental data are available for cell signaling networks. Accordingly, approaches with less required data, e.g. Boolean and Logic-based ODE (LDE), are prevalent for modeling large-scale signaling networks [36–39]. The other challenge in implementing a prior knowledge network model is its inability to predict signaling outside the pre-defined scope of the network. Hence, recently, researchers prefer to utilize hybrid approaches for modeling large-scale networks [40,41].

We previously developed a large-scale signaling network model of cardiac hypertrophy using logic-based differential equations [4]. This model was built from a wealth of prior knowledge and was validated against in vitro and in vivo rat data [4,42]. However, this model does not account for the network specificity in each context and needs revision to accurately predict myocyte response to inhibitory perturbations [4,23]. Moreover, emerging context-specific and patient-specific high-throughput data increases the need for systematic approaches to develop such network models.

In this study, we developed a systematic method to derive a hybrid context-dependent model for a signaling network, starting from its interaction graph and context-specific data. Employing the interaction graph of our previous hypertrophy network and context-specific data curated from the literature, we identified the key signaling reactions that regulate the cardiomyocyte response in four different contexts (hypertrophic agonists). More importantly, in developing a context-specific model for β-adrenergic-induced hypertrophy, we predicted and then experimentally validated previously unknown crosstalks between Ca^2+^-dependent and β-adrenergic non-classical pathways, CaMKII-Gβγ and CaN-Gβγ, which regulate hypertrophy network response in the context of β-adrenergic stimulation. These findings indicate the strength of the CLASSED approach in systematically predicting unknown context-specific network connections to provide a better understanding of a large-scale network response in different contexts.

## Materials and methods

### Ethics statement

All experimental procedures were conducted in compliance with the Guide for the Care and Use of Laboratory Animals published by NIH and approved by the University of Virginia Institutional Animal Care and Use Committee.

### Research pipeline

**Fig 1:**
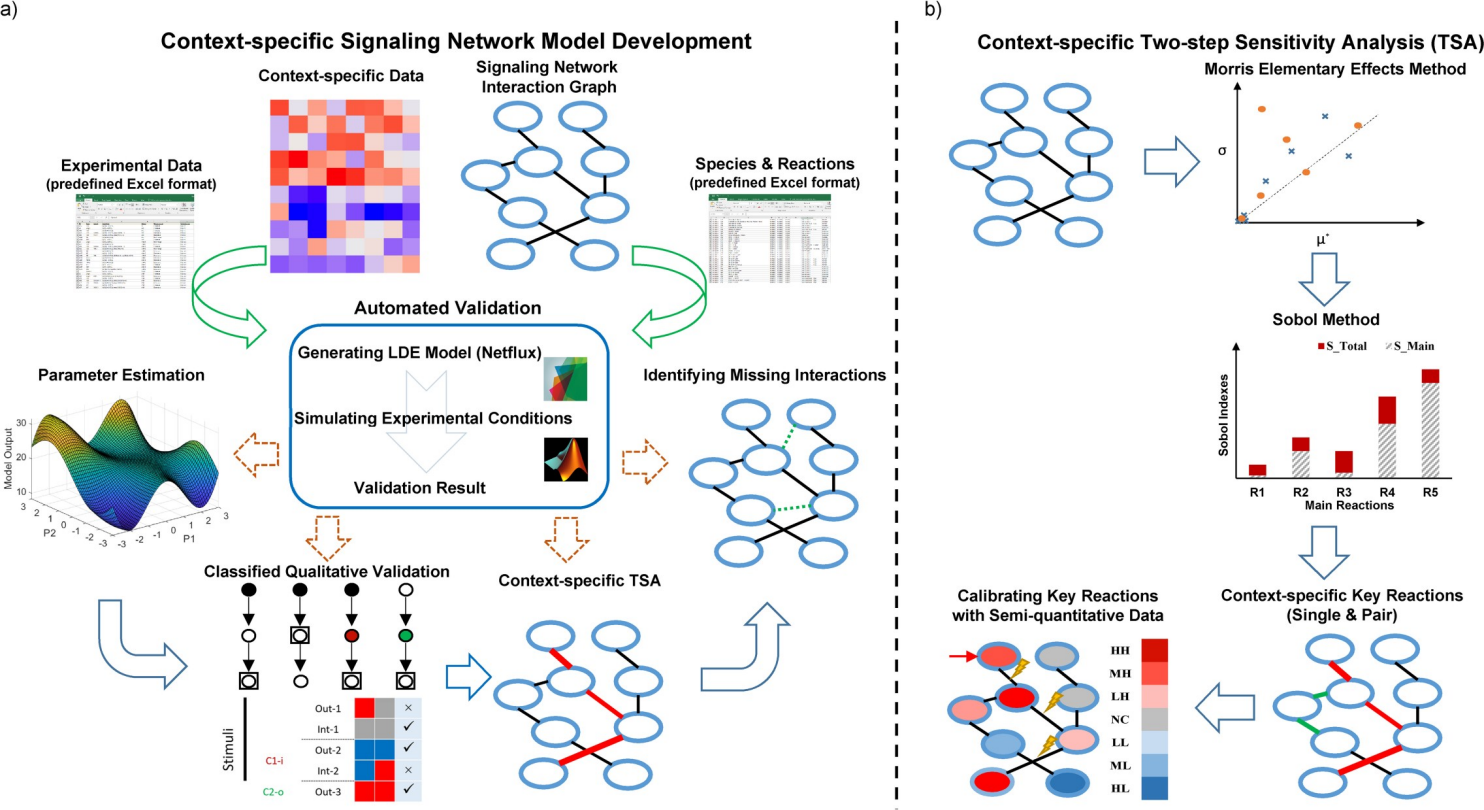
Pipeline of CLASSED approach. (A) Four-stage revision process to derive a hybrid context-specific model with an automated validation module as a core, and context-specific data and signaling network interaction graph as inputs. (B) Schematic of context-specific global sensitivity analysis (GSA). Two-step Morris-Sobol global sensitivity analysis identifies key reactions of model validity in each context and reduces the model dimension for calibration of main parameters with semi-quantitative data.

Fig 1 illustrates the pipeline of the CLASSED (Context-specific LArge-Scale Signaling nEtwork Development) approach to develop context-specific large-scale signaling network models. This approach employs 1) the interaction graph of a prior-knowledge network, and 2) the context-specific perturbation data, in predefined Microsoft Excel formats as inputs. Automated validation of the model is conducted by converting the interaction graph to a logic-based ODE (LDE) model through Netflux software (available at https://github.com/saucermanlab/Netflux) and simulating experimental conditions determined by context-specific data. We constructed a four-stage model revision process to develop a context-specific network model by implementing the automated validation module as a core (available at https://github.com/mkm1712/Automated_Validation). The default parameters of the model are estimated in the first stage from available qualitative data in all contexts. Next, the “Classified Qualitative Validation” module represents all validation data graphically in different contexts and data classifications, to demonstrate the heterogeneous performance of the model predictions. Performing a two-step Morris-Sobol global sensitivity analysis with the context-specific data in the third stage identifies the key signaling reactions, single and pairs, that affect model validity. This provides insight into context-dependency of the signaling network and reduces the model’s degrees of freedom to a practical range for subsequent calibration with semi-quantitative data. Furthermore, a single reaction deletion technique is presented as a parallel approach, with a lesser accuracy but a higher computational efficacy, to identifying key reactions in each context. Finally, all interactions between signaling pathways are explored via adding new reactions to signaling network nodes through the “OR” gates, and to network reactions through the “AND” gates. This analysis uncovers missing context-specific signaling interactions and crosstalks.

### Model construction

#### Prior-knowledge network

In this study, we made use of a hypertrophy network model (S1 Table) constructed by Ryall et al. [4] as the prior-knowledge network for our approach. The network topology was based on the 14 most established hypertrophic pathways from literature which links receptor inputs to transcription factors activities, cardiomyocyte size, and gene expression. The network contains 106 nodes, 191 reactions and 17 receptor inputs (S1 Fig).

#### Modified normalized-Hill logic-based differential equation approach

We applied a modified version of the normalized-Hill LDE approach to convert the interaction graph to a system of ordinary differential equations. Although these modifications are minor, we found that they are necessary for the method to be accurate in various cases. In this regard, we revised the formula of inhibition reaction to address two limitations of the previous version. As shown in S2A and S2B Fig, we expect a decrease in the weight factor (w) of an inhibition reaction increases the activity of its downstream effectors. This is not valid for the previous formulation. Moreover, setting the weight factor to zero in the “single reaction deletion” technique leads to incorrect baseline activities for a few signaling components such as GSK3β and foxo. Both issues have been addressed by updating the inhibition formula. It is noteworthy that in the previous studies, the weight factor was fixed to one in all cases and, consequently, their results were not affected by the mentioned limitations.

Furthermore, we modified the “AND” gate formula. As illustrated in S2C and S2D Fig, we divided the original “AND” gate output to the mean of its inputs to the power of n-1. Where n is the number of “AND” gate inputs. This adjustment increases the “AND” gate output’s sensitivity to the inputs’ perturbations when we have a large number of inputs (>3). It equalizes the order of magnitude between the output and inputs. S2C and S2D Fig illustrate the variations of an “AND” gate output in terms of its two inputs for the original and the modified formula. S2E Fig illustrates the sensitivity of model validation percent (for all data) to the *in silico* threshold for defining an increase or decrease in the model. While we have usually seen more than 10% relative changes in the activity of stimulated or inhibited species in experiments, capturing these variations by the model requires a lower threshold due to the reduced sensitivity of the model because of utilizing the same default parameters for all reactions and the existence of “AND” and “OR” gates with a large number of inputs in the network. As shown in S2E Fig, the reduced sensitivity of the model can be easily compensated by choosing an in silico threshold equal to 1% without a considerable effect on computational cost (6.6% increase in computational time in comparison with 10% threshold).

#### Model parameters

Eight default parameters regulate signaling nodes’ activity in the model. The first three are “EC_50_”, “n” (Hill-equation parameters) and “W_R_” (reaction weight) which are specific to intracellular signaling reactions. While “W_i_” adjusts the initial baseline activity of all inputs in a resting state, “W_e_” represents the elevated activity of a specifically stimulated input reaction, such as adding an agonist like isoproterenol. “Y_max_” determines the maximum activity of each node and is set to zero for the node inhibition. Finally, “Y_0_” and “tau” control the initial value and time constant of each node activity, respectively. The overexpression of a signaling node is simulated by setting its “Y_0_” and “tau” equal to “Y_max_” and 10^9^ (a large value), respectively. The single reaction deletion was performed by setting the weight factor “W_R_” of all signaling reactions to zero, one at a time.

#### Parameter estimation

We utilized the GlobalSearch optimization method of MATLAB Global Optimization Toolbox to find the optimized default parameters. We selected the GlobalSearch method due to its better performance in finding global minimum in terms of accuracy and repeatability in comparison with other methods such as pattern search and multistart. The GlobalSearch method [43] employs a scatter-search algorithm to create a set of trial points and heuristically chooses the time to execute a local optimization. The “fmincon” solver with the “sqp” algorithm has been selected for the GlobalSearch method. The repeatability of finding global minimum has been examined by repeating the estimation process with 100 random initial sets.

### Context-specific data collection

Context-specific data for the cardiac hypertrophy were manually curated from the literature. We obtained qualitative and semi-quantitative data at steady-state or an appropriate time point from research articles with similar cell lines, assay, and experimental conditions with a preference for data from neonatal rat ventricular myocytes. In total, 450 qualitative (all contexts) and 100 semi-quantitative (ISO-specific context) experimental data points have been obtained from 230 articles. The qualitative and semi-quantitative data are displayed in S2 and S3 Tables, respectively.

### Global sensitivity analysis

Two global sensitivity analysis methods were conducted sequentially to identify signaling reactions with the most influence on the model agreement with data in each context. In the first step, we utilized Morris’s elementary effects method which is considered a proper screening method for models with numerous parameters and/or when the computational cost for model simulation is relatively high. The “Sampling for Uniformity” strategy with oversampling size, input factor level, and trajectories’ number equal to 300, 8, and 16, respectively, has been applied to generate the Morris sampling data by EE sensitivity package developed by Khare et al. [44].

For the second step, the Sobol sensitivity analysis was employed. This variance-based method is considered a reference for evaluating other global sensitivity analysis approaches [45]. However, the Sobol method is not practical for models with many parameters owing to its extremely high computational cost. Hence, applying a screening approach to reduce the number of estimated parameters prior to Sobol analysis is indispensable for large-scale models. The Sobol-Jansen formula was utilized for estimating the Sobol indices [46]. Also, the 95% confidence intervals for Sobol indices comprising main, total and pair indices were computed by using the Bootstrap method with a resampling size equal to Sobol’s sample size and 100,000 replicates. Considering that no definite value is recommended for the Sobol’ sample size in literature, we performed the sensitivity analysis with different sample sizes from 1,000 to 100,000. The Sobol indices converged to their final value for sample size equal to and greater than 80,000 (4–5 million simulations) with all indexes greater than zero. Each run with sample size equal to 80,000 took eight hours on 20 parallel CPUs with 40 core for each CPU.

### Experimental approaches

#### Materials

The Janus Kinase 2 protein (JAK2) inhibitor AG 490, Mitogen-Activated Protein Kinase Kinase (MEK) inhibitor U0126, small molecule Gβγ inhibitor Gallein, Ca^2+^/calmodulin-dependent protein kinase II (CaMKII) inhibitor KN93, Calcineurin (CaN) inhibitor Cyclosporin A (CsA) and 4′,6-diamidino-2-phenylindole (DAPI) were all purchased from Sigma (USA). Isoproterenol hydrochloride (ISO) and Dimethyl sulfoxide (DMSO) were purchased from R&D Systems (USA). A monoclonal anti-sarcomeric actin antibody and Antibodies against p44/42 MAPK (ERK1/2), phospho-p44/42 MAPK (Thr202/Tyr204), α-Tubulin (DM1A) and secondary antibodies Alexa4 568 and 647 were purchased from Cell Signaling Technology (USA). ISO was dissolved in H2O and used at 10 μmol/L final concentration. Additional reagents were diluted in DMSO and added to the serum-free media with the following final concentrations: AG 490 (10 μmol/L), U0126 (1 μmol/L), Gallein (10 μmol/L), KN93 (1 μmol/L), and CsA (0.5 μmol/L). The final concentration of DMSO was lower than 0.3%.

#### Cell culture

Cardiac myocytes were isolated from 1–2-day-old Sprague-Dawley rats using Neomyts isolation kit (Cellutron, USA). The cells were cultured in plating media (66% low-glucose Dulbecco's modified eagle media (DMEM), 17% M199, 10% horse serum, 5% fetal bovine serum (FBS), 1% L-Glutamine, 100 U/mL penicillin, and 50 mg/mL streptomycin) at a density of 40,000 cells per well of a 96-well Corning CellBIND plate and 750,000 cell per well of a 6-well Corning CellBIND plate coated with SureCoat (Cellutron, USA). All medium solutions were buffered with HEPES (pH 7.5) to a final concentration of 20 mM. After 48 hours of incubation with daily media change, the medium was replaced with 0.1% FBS medium and the cardiac myocytes were serum-starved for 18 hours before treatment.

#### Western blot

Treated or control cardiac myocytes were washed with ice-cold PBS and digested with RIPA buffer [47]. After transferring the cell lysates to tubes, we pass lysates through the needles about 10 times and incubated it on ice for 20 minutes. The samples were centrifuged at 12,000 rpm for 20 minutes at 4°C, and the supernatant liquids were collected. Total protein concentration was quantified using the Pierce BCA protein assay kit (Fisher Scientific, USA). For each sample, 20 μg of total protein were electrophoresed on a 7.5% sodium dodecyl sulfate-polyacrylamide gel (Bio-Rad, USA) and then transferred to Polyvinylidene fluoride (PVDF) membranes (Sigma, USA). After blocking with 0.5X Odyssey (LICOR) blocking solution in TBST (Tris-buffered saline, 0.1% Tween 20) at room temperature for 1 hour, the membranes were incubated with primary antibodies (1:1000) overnight at 4°C. After extensive washing with TBST, proteins were detected with secondary antibodies (1:15000) IrDye 800CW and IrDye 680LT (Licor, USA) followed by quantification using a LICOR Odyssey system. The amounts of phospho-p44/42 MAPK and p44/42 MAPK were normalized to *α*-tubulin for all samples, and the significance of the difference between the treated and control samples was determined by two-tailed Student’s *t-test*.

#### Immunofluorescence and Imaging

Cell fixation after treatment was conducted with 4% paraformaldehyde (PFA) and followed by permeabilization with 0.2% Triton-X/TBS for 20 min and blocking by 1% BSA/TBS for 1 hour. Then, we incubated the cells with primary antibodies overnight at 4°C and subsequently blocked them by 2% normal goat serum (NGS) for 1 hour before incubating with appropriate secondary antibodies. The cells were stained with DAPI for visualizing nuclei (blue), monoclonal anti-α-Actinin antibody (red) for cardiac myocytes, and phospho-p44/42 MAPK antibody (green) for ERK1/2 activity. The fluorescence images were captured using the Operetta High Content Screening System (Perkin Elmer, USA). The central images were analyzed via MATLAB.

## Results

### Estimation of default parameters for myocyte-specific context

**Fig 2:**
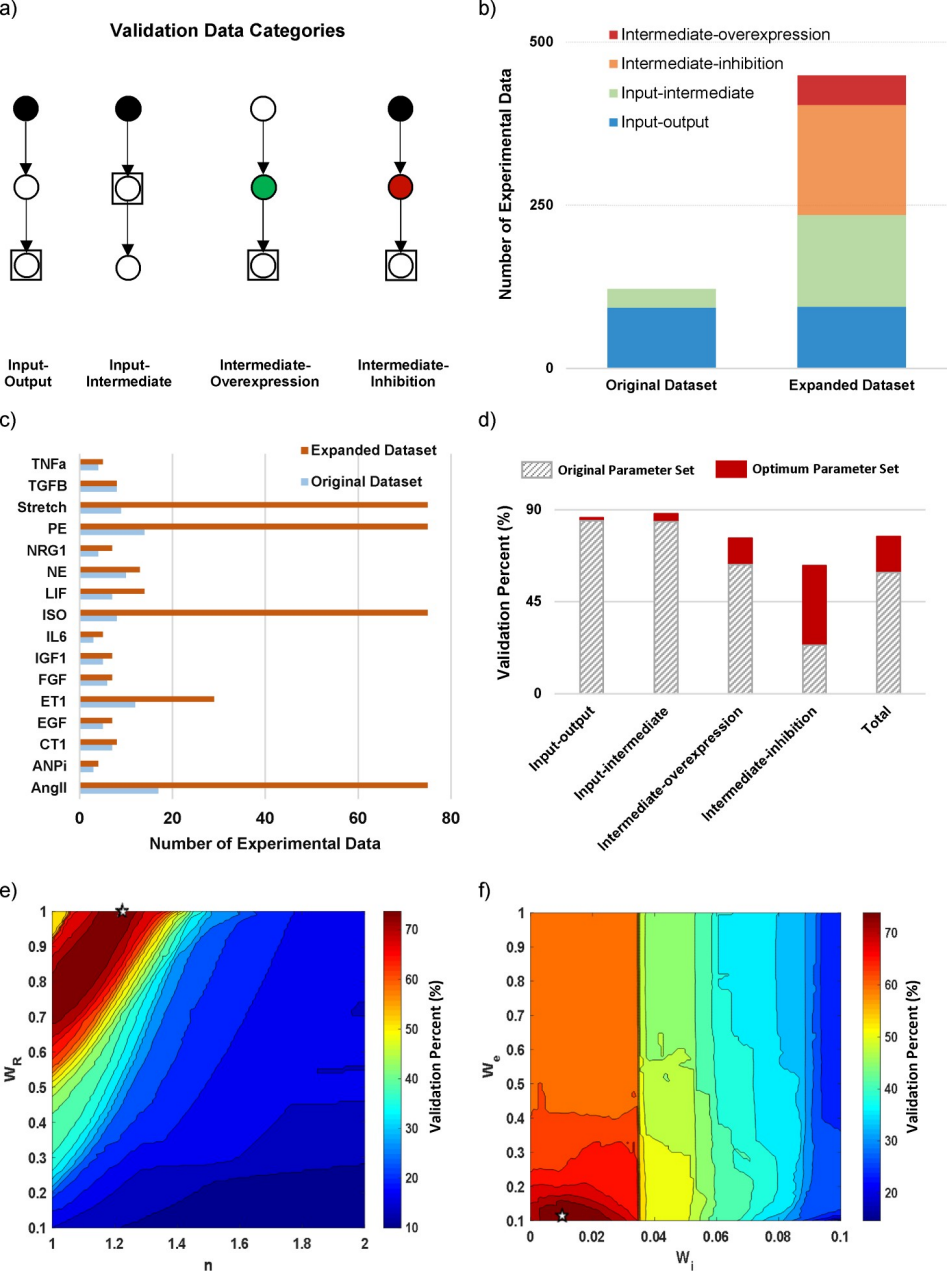
Estimating default parameters enhances model prediction accuracy in an expanded data compendium covering four validation categories. (A) Classification of myocyte-specific data in four classes based on the function of measured and perturbed signaling nodes in the network. (B) Comparison of the original [4] and expanded datasets in terms of frequency of each data class. (C) Data distribution in the original and expanded datasets regarding the context (hypertrophic agonist). (D) Comparison of the model prediction accuracy in all data classes before and after estimation of default parameters. (E, F) Variations of model validation percent in terms of four default parameters of the model (n, W_R_, W_i_, W_e_). In each figure, the non-variant parameters were fixed to optimum values. The white star shows the optimum parameter set.

Qualitative observations representing variations in signaling network nodes in response to hypertrophic agonists were obtained from the literature at the steady-state, or suitable time points for nodes with transient activity. While our logic-based differential equation approach predicts signaling dynamics, due to lack of sufficient kinetic experimental data we focused mostly on steady-state conditions to study causal interactions in cardiac hypertrophy signaling network. All observations were recorded as an increase, decrease, or no change according to their published statistical significance in comparison to the control group in each study. Data were categorized in four classes: Input-Output, Input-Intermediate, Intermediate-Inhibition, and Intermediate-Overexpression (Fig 2A). Validations in the original manuscript reporting this model were from only Input-Output and Input-Intermediate data types [4]. But given that many context-specific responses originate from mediators of signal transduction, we found it important to expand the validation dataset in terms of data classes and numbers (Fig 2B).

In this study, we acquired data for 16 different agonists but largely focusing on four established hypertrophic agonists as the studied contexts involving isoproterenol (ISO), phenylephrine (PE), angiotensin II (Ang II) and biomechanical stretch. The number of data for each context is displayed for the original and expanded datasets in Fig 2C. Each of studied contexts includes 75 qualitative experimental data points. Next, by importing the model interaction graph and expanded dataset to the automated validation module, we computed the model validation percent. The validation module simulates each defined experimental condition; categorizes the simulation results based on their percent of changes from control with 1% change as the threshold; and computes the percent of agreement between model predictions and the matched biochemical measurements.

As shown in Fig 2D, the hypertrophy model with original default parameters from Ryall et al. study [4] predicts the data on Input-Output and Input-Intermediate categories quite well, with more than 80% correct prediction in both classes. Its performance was lower for Intermediate-Overexpression data, with ~60% accuracy. In the Intermediate-Inhibition class of data, we observed a low prediction accuracy (24%).

To test whether the poor accuracy could be explained by non-optimal default parameters, we performed a parameter estimation. The parameter’s values in the original model [4] had been set based on the default values from beta-adrenergic signaling model [39]. In this study, we estimated the four default parameters of the model including Hill coefficient “n”, reaction weight “W_R_”, initial input weight “W_i_” and experimental weight “W_e_” based on the hypertrophic experimental data. As shown in Fig 2D, estimating these default parameters significantly enhances the model prediction accuracy particularly in the Intermediate-Inhibition class of data from 24% to 63%. Also, we observed improvements in Intermediate-Overexpression class with more than 75% correct predictions, similar to the total model validation percent. To examine the extent to which uncertainty in parameters affects the model prediction accuracy, the variations of model validation percent in terms of estimated parameters have been illustrated in Fig 2E and 2F. While a gradual variation in model predictive power was seen for the pair “W_R_”, “n” in the diagonal direction, its variation for the pair “W_e_”, “W_i_” is horizontally and stepwise. This result indicates that while the change in the agonist activity “W_e_” has the lowest effect on model validity, elevation of signaling nodes’ basal activity significantly deteriorates the model validity. Furthermore, variations of estimated parameters in the neighborhood of the optimum point with 77% agreement with data have no significant impact on the model validation percent.

### Identifying candidates for context-dependent model revision

**Fig 3:**
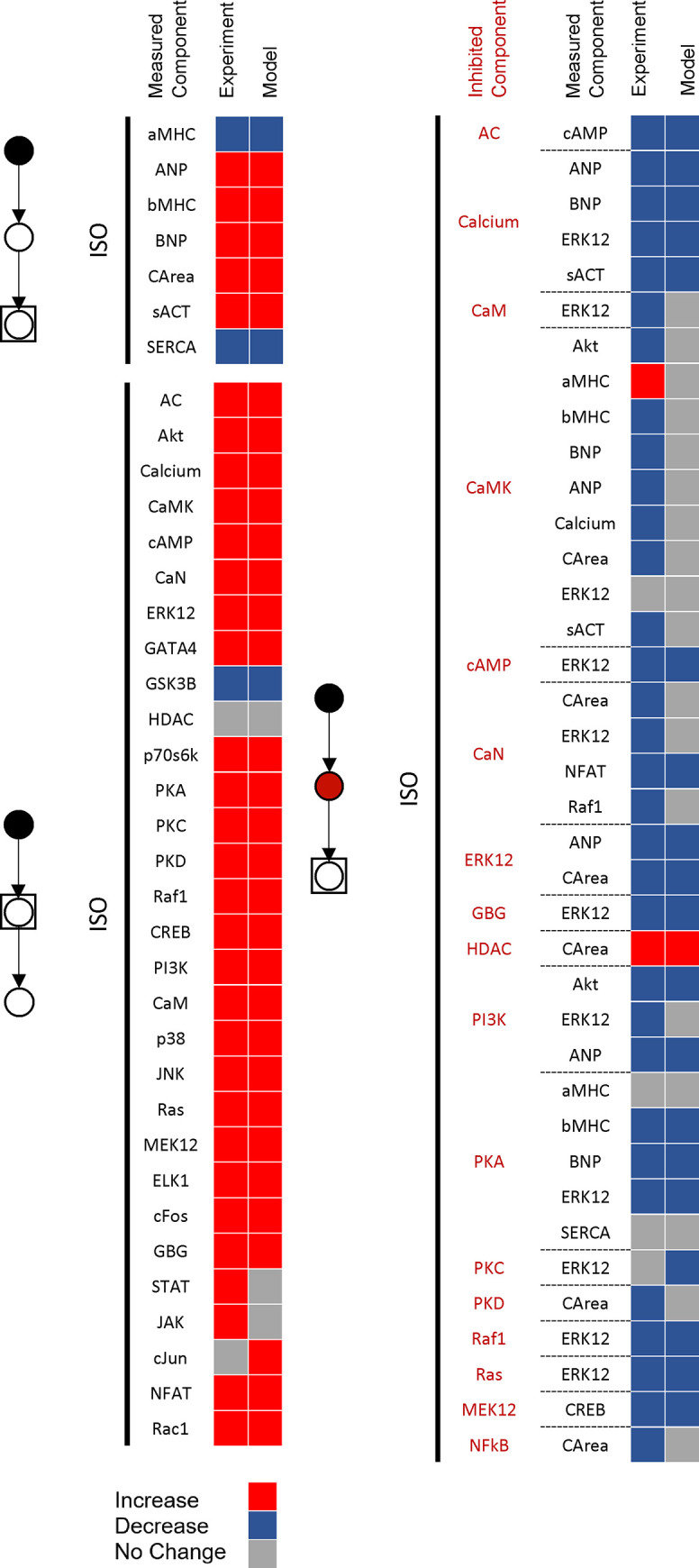
Classified qualitative validation reveals incomplete parts of the ISO-specific network. Comparison of model predictions with 75 experimental data points in three classes of data including input-output (7/7 correct prediction), input-intermediate (27/30 correct prediction), and intermediate-inhibition (22/38 correct prediction) after isoproterenol stimulation. The red, blue, and gray boxes illustrate increase, decrease, and no change, respectively. In the model, changes in the measured node activity greater than +1% or smaller than -1% have been considered as an increase or decrease, respectively. In experimental data, statistically significant changes in comparison with the control have been considered as an increase or decrease based on the direction of changes.

After calibrating the four default parameters based on qualitative data of all contexts, we employed validation module’s output to illustrate validation data in regard to the contexts and data classes. Fig 3 displays the classified qualitative validation of the model in ISO-specific context. As can be seen, prediction of network outputs activity (blue nodes in S1 Fig) in response to ISO had no disagreement with experimental data within the input-output class (7/7 correct prediction). Also, validations where intermediate nodes were measured as the output had similarly a high agreement with data (27/30 correct prediction). In ISO-specific context, the model had lower efficacy in Intermediate-Inhibition data class (22/38 correct prediction), and CaMKII- and CaN-related data (2/13 correct prediction). Thus, CaMKII and CaN seemed to be the main sources of inefficiency in the model structure, which we later targeted for model revision. Similar results can be seen in other contexts in regard to the model prediction accuracy for different data classes (S3 Fig). However, we observed different sources of inefficiency, in addition to CaMKII and CaN, in other studied contexts such as NFκβ and RhoA in Ang II and stretch contexts, respectively [48].

The main reason for considerable disagreement in the Intermediate-Inhibition class is the context-dependency of the measured data. This reflects its impact on model predictions in Intermediate-Inhibition class. This context-specific response could be due to multiple reasons including inaccurate reaction parameters, erroneous combinatory logic, and incomplete model structure. Inaccurate parameters such as reaction weight could be influencing the individual contributions of each pathway to the observed outputs. In the current state of the model, all reactions have equal reaction weights, when in reality some reactions may be exerting more or less of an influence than others [12,49]. The model could become more sensitive to a given inhibition if certain reactions were given higher weight than others, which is applied to the model in the third stage. Incorrect reaction logic could also lead to a greater insensitivity of model to nodes inhibition, as combinations with "AND" gates are generally more sensitive to a node inhibition than "OR" gates. The low predictive power may also be the outcome of an incomplete model structure, where new reactions are required to correctly predict the experimental outcomes. We address this issue in the final stage of CLASSED approach.

### Reactions required for context-specific agreement with experimental data

One of the challenges in analyzing large-scale networks is to systematically determine how modification in individual reactions will affect the comprehensive set of validations. In the same vein, it is complicated to find out which reactions are responsible for the model's inconsistency with experimental data. To address this issue, firstly, we employed automated validation module to test the local effects of each reaction on model validation percent by revalidating the model with context-specific data following systematic, one-by-one removal of each 174 intermediate reactions in the network for each context.

**Fig 4:**
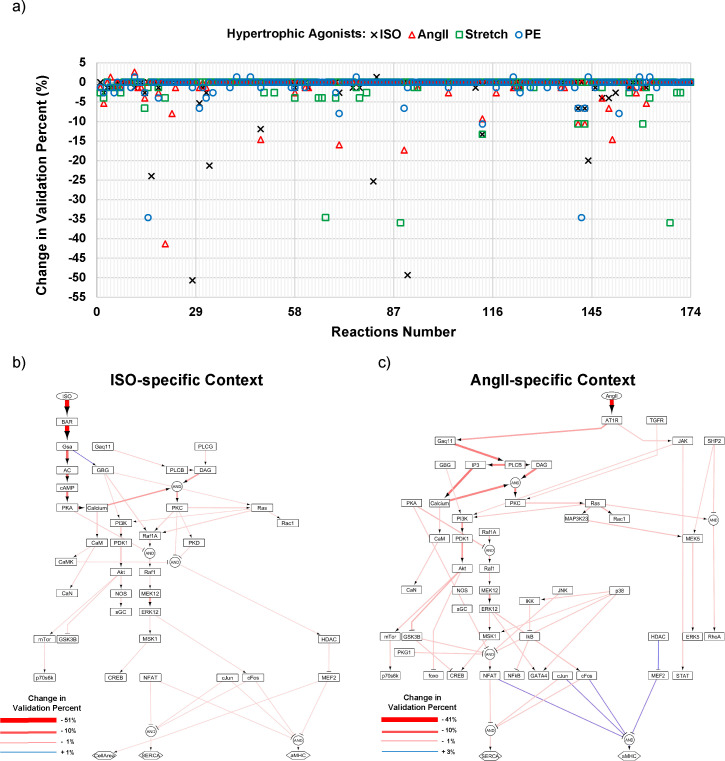
Single-reaction deletion identifies and categorizes context-specific and shared key reactions. (A) Changes in model validation percent after one-by-one removal of each 174 intermediate reactions in the hypertrophy network for ISO (cross), PE (circle), AngII (triangle), and stretch (square) contexts. (B) The key reactions in ISO-specific and (C) AngII-specific contexts have been visualized on the hypertrophy network map. The thicker and bolder red, or blue arrows illustrate more decrease, or increase in validation percent, respectively, after removing each reaction.

Fig 4A illustrates the results of single reaction deletion technique for four studied contexts in the hypertrophy network. Based on the results, three categories of reactions can be recognized in each context. In the “non-sensitive reactions” category, deleting a reaction has zero or a small positive effect on model validity. Interestingly, the majority of hypertrophy network reactions have been categorized as non-sensitive reactions, which can be explained by several intrinsic properties of the hypertrophy network. As shown in S1 Fig, there are many redundant parallel pathways in the hypertrophy network, which lead to the insensitivity of model to the elimination of a single reaction. Applying the same default parameters for all reactions also intensifies this insensitivity. Moreover, there exist several hubs in the hypertrophy network like Ras, PI3K, p38, and ERK1/2 with many inputs or outputs which transduce the signal regardless of deleting a single reaction. Hence, applying more comprehensive approaches like global sensitivity analysis which consider the variations of all reaction parameters at the same time seems to be essential to scrutinize the role of each reaction in the overall response of the network.

The “direct reactions” category includes the reactions which are specific to each context and have little or no effect on other contexts. This category mostly comprises the direct downstream reactions of the main receptor for that context. On the other hand, the “shared reactions” category involves reactions that are crucial for multiple contexts and could play a switching role in different context-dependent responses. To determine key reactions, we ranked the reactions in terms of change in the validation percent after removing each reaction. Revision of a model should be done with caution when modifying high score reactions because these reactions are required for a large number of correct predictions. Conversely, non-sensitive reactions when removed from the model indicate a non-sensitive area of the network and could be targeted for model reduction. Under the context of ISO stimulation, single reaction deletion affected model accuracy only for 40 out of 174 model reactions.

To examine how key reactions differ across contexts, the results of the single reaction deletion were visualized on the hypertrophy network map for ISO-specific and AngII-specific contexts in Fig 4B and 4C, respectively. As expected, upstream reactions in the classical β-adrenergic signaling pathway [50] from βAR to protein kinase A (PKA) have the highest effect on model validity in ISO-specific context and would be categorized in the “direct reactions”. Similar results could be obtained for the AngII-specific context in which upstream reactions from AngII receptor (AT1R) to Inositol trisphosphate (IP3) have the highest score. In these two contexts, reactions such as PI3K = >PDK1 = >Akt and MEK12 = >ERK1/2 (ERK1/2 pathway in general) have significant impacts on both contexts and identified as “shared reactions”. These reactions might be the potential targets for further experiments to understand distinct myocyte responses after ISO and angiotensin II stimulations [51,52]. The results of single reaction deletion technique for other contexts, PE-specific and Stretch-specific, are depicted in S4A and S4B Fig. Comparing all contexts, S4C Fig**,** shows that more shared reactions are involved in the cardiac hypertrophy signaling in PE-specific and AngII-specific contexts than ISO-specific and Stretch-specific contexts.

Whereas the single reaction deletion method is easily interpretable, a limitation is that it does not account for nonlinear interactions between reactions in the hypertrophy network. Therefore, we applied a two-step global sensitivity analysis to detect the key reactions for hypertrophy model validity (for details see description in method's section). In this stage, we selected EC_50_ as a variable of sensitivity analysis due to the following reasons: 1) the weight factor “W_R_” was estimated before in the first stage to enhance the model accuracy in predicting myocyte-specific data (EC_50_ was fixed); 2) based on the LDE formulation, EC_50_ is the most influential parameter of each reaction [39] and more diverse reactions could be obtained by using EC_50_ than W_R_ (S5 Fig) as a variant parameter of the Morris method which provides identification of transactivation in each context; and 3) unlike “W_R_”, using EC_50_ as a variant parameter predicts similar key reactions for each context regardless of choosing different model outputs (blue nodes in S1 Fig) or model validation percent as the objective function which provides robust results.

**Fig 5:**
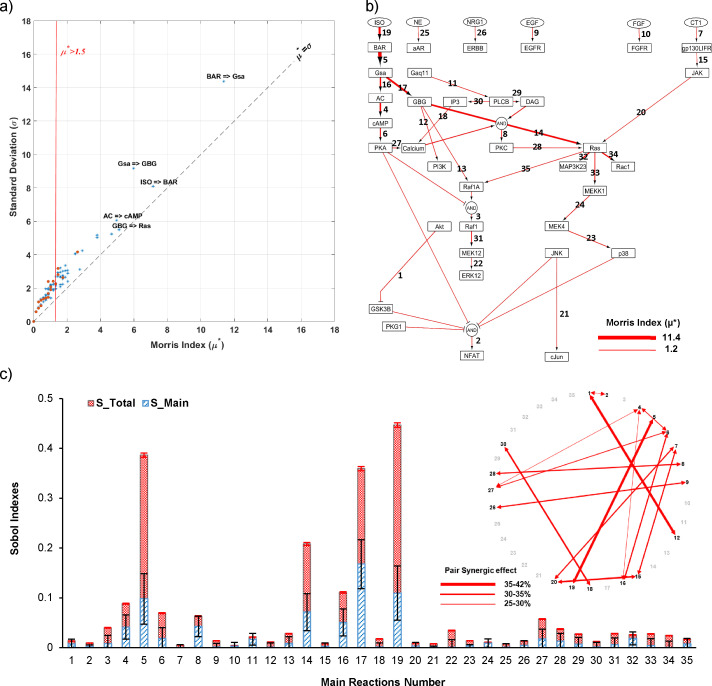
Two-step Global sensitivity analysis identifies key single and pair reactions in an ISO-specific context. (A) Morris sensitivity analysis result including Morris index (μ*) and standard deviation (σ) illustrates important and non-important reactions in the ISO-specific context. Greater Morris index (μ*) demonstrates more influence on model validation percent. Larger σ to μ* ratio (above μ* = σ diagonal line) demonstrates a more nonlinear effect on model prediction accuracy which implies on more interactions with other network reactions. The orange dots and blue stars illustrate monotonic and non-monotonic effects of each reaction on model validation percent, respectively. The vertical red line indicates the significance level for identifying non-important reactions. (B) Visualization of important reactions in ISO-specific context based on their ranks from Morris sensitivity analysis. The thicker red arrows illustrate the larger Morris index (μ*). (C) Sobol sensitivity analysis reveals importance of nonlinear interactions between network signaling reactions in ISO-specific context. The Total (checked red bars) and Main (hashed blue bars) Sobol indexes and pair synergic effects are illustrated for important reactions (35/174) in ISO-specific context obtained from Morris sensitivity analysis. The error bars represent 95% confidence interval of a mean. More difference between the Total and Main indexes demonstrates a more nonlinear effect. The pair synergic effect displays the ratio of the second-order effect of each reaction pair to the sum of higher-order effects of its reactions.

The results of Morris sensitivity analysis for ISO-specific context are displayed in Fig 5A and 5B. Since the level of significance for μ* is case-dependent, we assumed μ* equals to 1.5 as a threshold to obtain key reactions in ISO-specific context. While this selection could be changed based on the model structure and the number of parameters (e.g. 1.2 for other contexts), we selected this value to obtain a range of 25 to 40 effective reactions considering that these reactions would be utilized as the inputs of Sobol sensitivity analysis. Considering higher threshold results in the reduced number of inputs for Sobol sensitivity analysis and higher chance of missing an important reaction or nonlinearity effect between reactions. On the other hand, lowering the threshold increases the number of effective reactions which results in a much higher computational cost without gaining much information about key reactions in most cases. Therefore, choosing an appropriate value for the level of significance in Morris results is case-dependent. As shown in Fig 5B, 35 out of 174 reactions have a considerable effect (μ* > 1.5) on model validation percent in ISO-specific context. Interestingly, all reactions were above the diagonal line (μ* = σ) in Fig 5A, which means that they have a dominant interaction (nonlinear) effect on model accuracy. In other words, these reactions primarily influence cell signaling through combination with other network reactions. Thus, when regulating these reactions experimentally, for example to find a treatment, we should consider controlling their pair reactions, too. As the orange dots illustrate the reactions with monotonic effect, most of the key reactions in ISO-specific network have a non-monotonic effect on model agreement with data. This means that we could observe different responses in regulating a specific reaction in β-adrenergic-induced hypertrophy and there is an optimal range for the concentration of the drug. In contrast to the findings of single reaction deletion technique, Morris analysis indicated that the β-adrenergic non-classical pathways [50] play the main role in regulating the overall model response in ISO-specific context. This contradiction seems to be due to the importance of nonlinear interactions in β-adrenergic hypertrophy network. Morris sensitivity analysis’ results for other studied contexts (AngII, PE, stretch) are depicted in S6 Fig. As can be seen, there are more reactions under the diagonal line in other contexts which indicates reactions with higher direct effect on cell overall response. These reactions could be the potential targets for regulating cell response with less concern of interference from other signaling reactions.

In the second step, we fixed the varying parameter (EC_50_) of all non-important reactions identified by Morris sensitivity analysis and performed Sobol sensitivity analysis on the reduced version of the model. By employing the Sobol-Jansen formulation, we obtained Monte Carlo estimation of total, main (first-order), and second-order Sobol indices for each reaction. As shown in Fig 5C, ISO = >βAR and Gsα = >Gβγ reactions have the highest total (0.44) and main (0.17) effects on model validation percent in ISO-specific context, respectively. Regarding the synergy effect, pairs of (ISO = >βAR, βAR = > Gsα), (Gβγ = >PI3K, Akt = >GSK3β), (gp130LIFR = >JAK, JAK = >Ras) and (PLCB = >IP3, IP3 = > Calcium) have the largest relative synergy in the ISO-specific contexts with more than 30% synergy effect.

These results are consistent with Morris method outcomes and confirm the necessity of utilizing global sensitivity analysis to predict context-specific key reactions in large-scale signaling networks. The key reactions of model agreement with data in each context are shown in S7 Fig. Finally, after identifying the key single and pair reactions in ISO-specific context, we developed an ISO-specific model of cardiac hypertrophy by calibrating the EC_50_ of 12 main reactions making use of both qualitative data and semi-qualitative data (S3 Table) in ISO-specific context. The S8 Fig illustrates enhancement of model prediction accuracy (from 47% to 60%) in ISO-specific context.

### Predicting new crosstalks in ISO-specific context

In general, developing a context-specific model from the prior-knowledge model needs revision of both parameters and structure of the model. In the previous section, we revised the prior model key parameters to obtain an ISO-specific model for cardiac hypertrophy. But there exist some experimental observations in β-adrenergic-induced hypertrophy that the calibrated model still cannot predict (S8 Fig). Therefore, we explored all possible new crosstalks in the network by systematic, one-by-one adding of new interactions between network nodes through the “OR” gate. We also explored adding new interactions from each network node to the existing reactions through the ‘AND” gate. Similar to the results of classified qualitative validation, we identified CaMKII and CaN as two source nodes with the highest potential in increasing the model agreement with data. In the ISO-specific context, the top-ranking reactions which improved model agreement with experimental data were new interactions from CaMKII and CaN to the upstream nodes of the Ras/Raf/MEK/ERK1/2 pathway.

**Fig 6:**
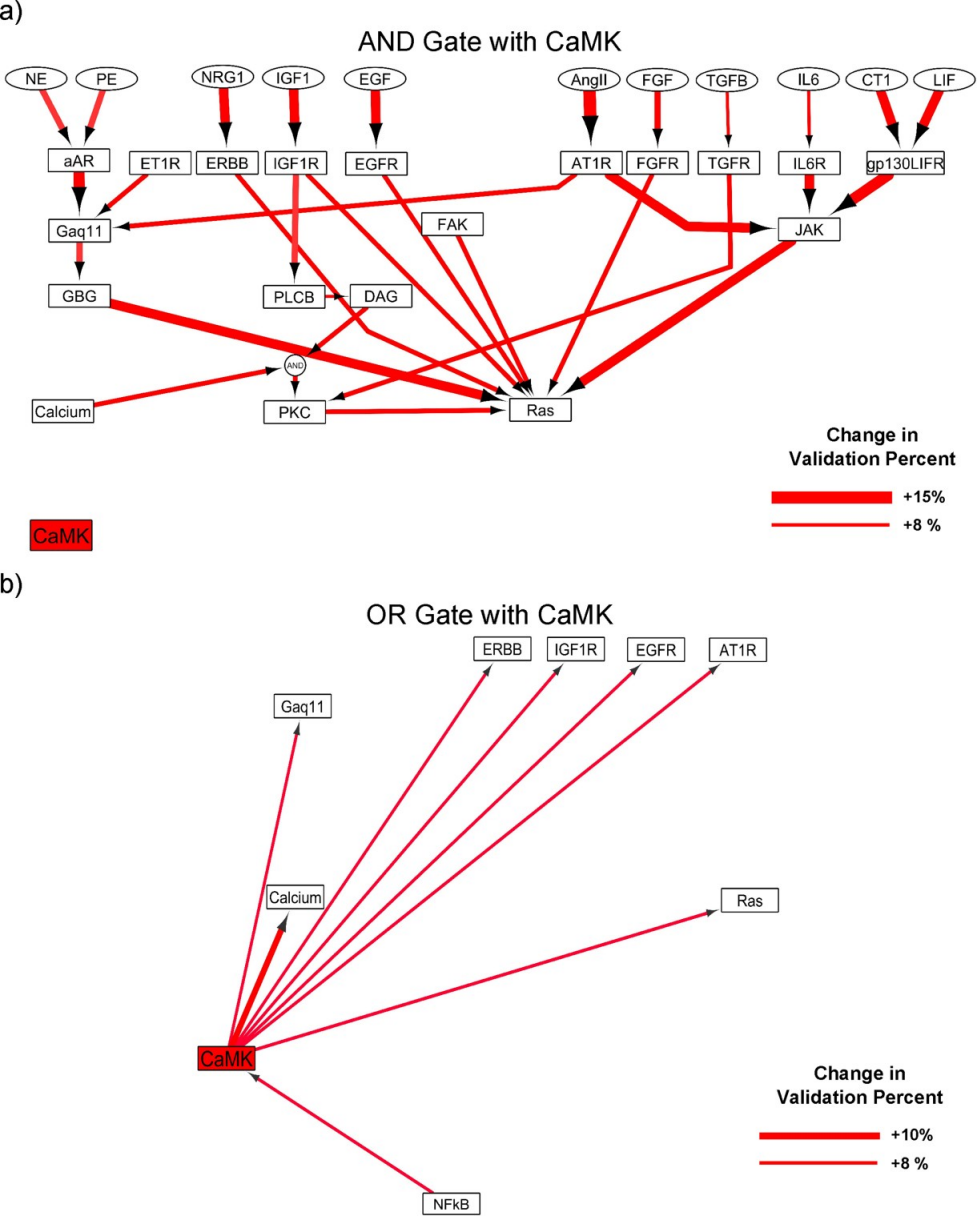
Exploring possible interactions nominates CaMKII as the key signaling node for missing crosstalks in ISO-specific network. (A) Model predictions for crosstalks between CaMKII and network reactions through the “AND” gate. Each arrow represents a reaction that by adding an “AND” gate from CaMK to that reaction, we observed a significant improvement in model accuracy. (B) Model predictions for crosstalks between CaMKII and network nodes through the “OR” gate. The thicker red arrows illustrate reactions with higher positive effect on the validation percent in ISO-specific model.

Fig 6 illustrates the top nominations for crosstalks in ISO-specific context by different logic gates associated with CaMKII. As can be seen, individual crosstalks with the “AND” gate lead to higher increase (maximum +15%) in model prediction accuracy compared with crosstalks with the “OR” gate (maximum +10%). The model predicts that combinations of CaMKII with the upstream reactions of the Ras have the most positive effect on the model validation (8–15% increase). This prediction suggests a contribution of CaMKII in the activation of β-adrenergic non-classical pathways. Among the model predictions, reactions associated with JAK and Gβγ have the highest ranks. Among the combination of CaMKII with other network nodes through the “OR” gate, the positive feedback from CaMKII to Calcium has the highest rank. The other predictions indicate the role of CaMKII in the transactivation of other hypertrophic pathways in β-adrenergic-induced hypertrophy.

Exploring all possible “AND” gates in the hypertrophy network (18444 cases) showed that for the ISO-specific network, 12.1% of new interactions decreased model validation percent. 5.4% of cases increased model validation percent, of which only 1.4% resulted in improving prediction of more than one data point of 75 validation data points. As expected, 82.5% of cases did not affect model predictions. For the “OR” gate in ISO-specific context (11236 cases), 39% of cases resulted in a decrease in model validation percent. 2.7% of cases increased model validation percent which only 1.2% resulted in improving prediction of more than one data point. 58.3% of “OR” gate cases did not affect model validation percent.

In addition to the CaMKII, the model predicts a significant increase (8–9%) in model validation by adding new interactions through “AND” gate from CaN to the upstream reactions which activate Ras in the hypertrophy network (S9A Fig). Also, after adding the positive feedback from CaMKII to Calcium which was predicted by the model, we repeated the adding reaction process with the modified model and observed that interactions between CaN and other reactions through the “AND” gate have the highest rank in enhancing model validation percent (5–7%), see S9B Fig.

### Validation of new crosstalks in β-adrenergic-induced hypertrophy

In the previous section, the model predicted three main crosstalks in ISO-specific context. First, a positive feedback from CaMKII to calcium through an “OR” gate has been predicted. This feedback signal has been verified by numerous previous studies on cardiac electrophysiology and calcium signaling [53–55]. For instance, Lee et al. [56] demonstrated that CaMKII is involved in the facilitation of L-type calcium channels in cardiac myocytes. Moreover, a review article by Grimm and Brown [57] describes CaMKII targets in β-adrenergic signaling, explains regulation of cardiac calcium signaling by CaMKII through a positive feedback with mediators like Ryanodine receptor, Phospholamban, and L-type Calcium channels [58].

The other two predictions of our model indicate the role of CaMKII and CaN in the activation of non-classical β-adrenergic pathways after ISO stimulation. While numerous studies have examined either the role of CaMKII and CaN in cardiac hypertrophy [13,59] or Ras/Raf/MEK/ERK1/2 pathway in cardiac hypertrophy [60–62], very few have examined cross-talk between calcium and MAPK pathways for cardiac hypertrophy. Zou et al. [63] investigated ISO-induced ERK1/2 phosphorylation in cultured cardiomyocytes, which they found to require CaN but not CaMKII. Sanna et al. [64] founded that calcineurin-NFAT and MEK1-ERK1/2 signaling pathways are interdependent and by forming a complex in cardiac myocytes coregulate the hypertrophic response.

**Fig 7:**
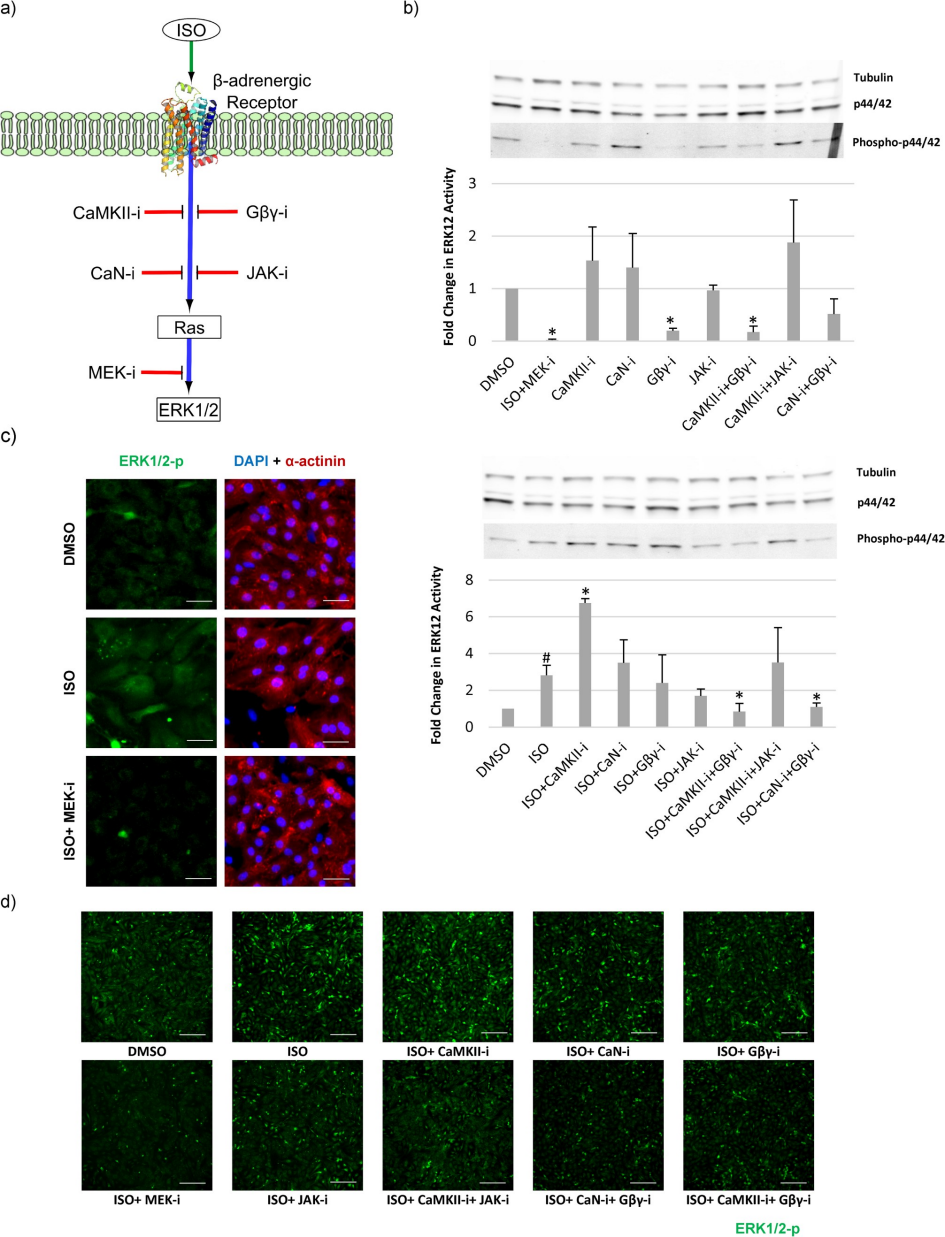
Inhibition of CaMKII-Gβγ or CaN-Gβγ suppresses ISO-induced ERK1/2 activity. (A) Effects of pretreatment for 30 min with five inhibitors including JAK-i (AG490), CaMKII-i (KN93), CaN-i (CsA), Gβγ-i (Gallein), and MEK-i (U0126) were examined on basal and ISO-induced ERK1/2 activity. (B) Upper panel shows western blotting of ERK1/2 phosphorylation after pretreatment with inhibitors in comparison with control (DMSO). Lower panel shows ERK1/2 phosphorylation after ISO stimulation with pretreatment with same inhibitors. *represents statistically significant change (p<0.05) in comparison with control (DMSO) for the upper panel, or with ISO in the lower panel. # represents significant change (p<0.05) compared with control (DMSO) in the lower panel. Data were collected from 3 independent experiments (Mean±SEM). (C) Immunofluorescence images show ISO-induced ERK1/2 phosphorylation and its suppression by MEK inhibitor in myocytes (alpha-actinin positive) and fibroblasts (alpha-actinin negative). The scale bar is 50 μm (D) Immunofluorescence imaging of ERK1/2 phosphorylation shows increase of ERK1/2 phosphorylation after CaMKII inhibition with KN93 and its suppression after pretreatment with Gβγ+CaMKII and Gβγ+CaN combined inhibitions. The scale bar is 200 μm.

To experimentally validate the model-predicted crosstalks between MAPK signaling and CaMKII/CaN, we assessed the effects of CaMKII, CaN, JAK2, MEK12 and Gβγ inhibition on ISO-induced ERK1/2 phosphorylation in primary neonatal rat cardiomyocytes (Fig 7A). Among nodes upstream of Ras, we chose to target Gβγ due to its predicted central role in ISO-specific context (Figs 5C, 6A and S7A) and JAK2 due to its mediatory position for high rank reactions (Fig 6A). ERK1/2 phosphorylation was assessed by Western blotting with or without isoproterenol for 10 minutes after 30 min pre-treatment with specified inhibitors. As expected, ISO increased ERK1/2 phosphorylation, and pretreatment with MEK12 inhibitor suppressed ISO-induced ERK12 phosphorylation (Fig 7B). Without ISO, only Gβγ inhibition with Gallein, and its combination with CaMKII inhibition (KN93), significantly diminished the basal level of ERK phosphorylation (80% decrease) compared to the control (DMSO). With ISO treatment, CaMKII inhibition by KN93 increased ERK1/2 phosphorylation significantly (140% increase in comparison with ISO), inhibition with Gβγ had no statistically significant effect, and combination of CaMKII and Gβγ inhibition significantly diminished ERK1/2 phosphorylation (70% decrease in comparison with ISO). These data indicate that combined CaMKII and Gβγ activation are required for full ISO-induced ERK1/2 phosphorylation. These experimental observations validate the model prediction of a missing crosstalk which connects CaMKII to Gβγ = >Ras reaction via an “AND” gate (Figs 6A and S9A).

Calcineurin (CaN) inhibition also modified ISO-induced ERK1/2 phosphorylation, but to a lesser degree. CaN inhibition alone had no significant effect on ISO-induced ERK1/2 phosphorylation, but combined CaN and Gβγ inhibition reduced ERK1/2 activity significantly by 60% in comparison with ISO. Thus, like CaMKII, CaN contributes with Gβγ to enhance ERK1/2 activity.

The western blotting is not usually measuring only cardiomyocytes’ response because protein lysates comprise both cardiomyocytes and non-cardiomyocytes (mostly fibroblasts). Therefore, we repeated the above perturbations but measured ERK1/2 localization and phosphorylation by immunofluorescence, which can discriminate between responses in cardiomyocytes and non-cardiomyocytes (Fig 7C and 7D). In cardiomyocytes, ISO stimulation for 10 min increased ERK1/2 phosphorylation primarily in the perinuclear area. In contrast, fibroblasts exhibited much stronger ERK1/2 phosphorylation, consistent with nuclear translocation (Fig 7C). Pretreatment with MEK12 inhibitor suppressed ISO-induced ERK1/2 activity in both cardiomyocytes and fibroblasts. We further examined the influence of pretreatment with different inhibitors on ISO-induced ERK1/2 activity in both cardiomyocytes and fibroblasts by immunofluorescence (Fig 7D). Response of ERK1/2 phosphorylation in cardiomyocytes was qualitatively similar to the results from western blotting. Both cardiomyocytes and fibroblasts exhibited the strongest ERK1/2 phosphorylation with combined ISO stimulation and CaMKII inhibition by KN93. Like in western blotting, we found significant decrease in ISO-induced ERK1/2 activity in the combination of Gβγ inhibitor with CaMKII or CaN inhibitors. The results further support the finding of crosstalk between calcium and Gβγ signaling in both cardiomyocytes and fibroblasts. To quantify the difference between cell types in ISO-induced ERK1/2 response, we thresholded images based on alpha-actinin expression. As illustrated in S10 Fig, ISO-induced ERK1/2 activation was much stronger in fibroblasts than cardiomyocytes, but both cells had responses qualitatively consistent with the western blotting.

**Fig 8:**
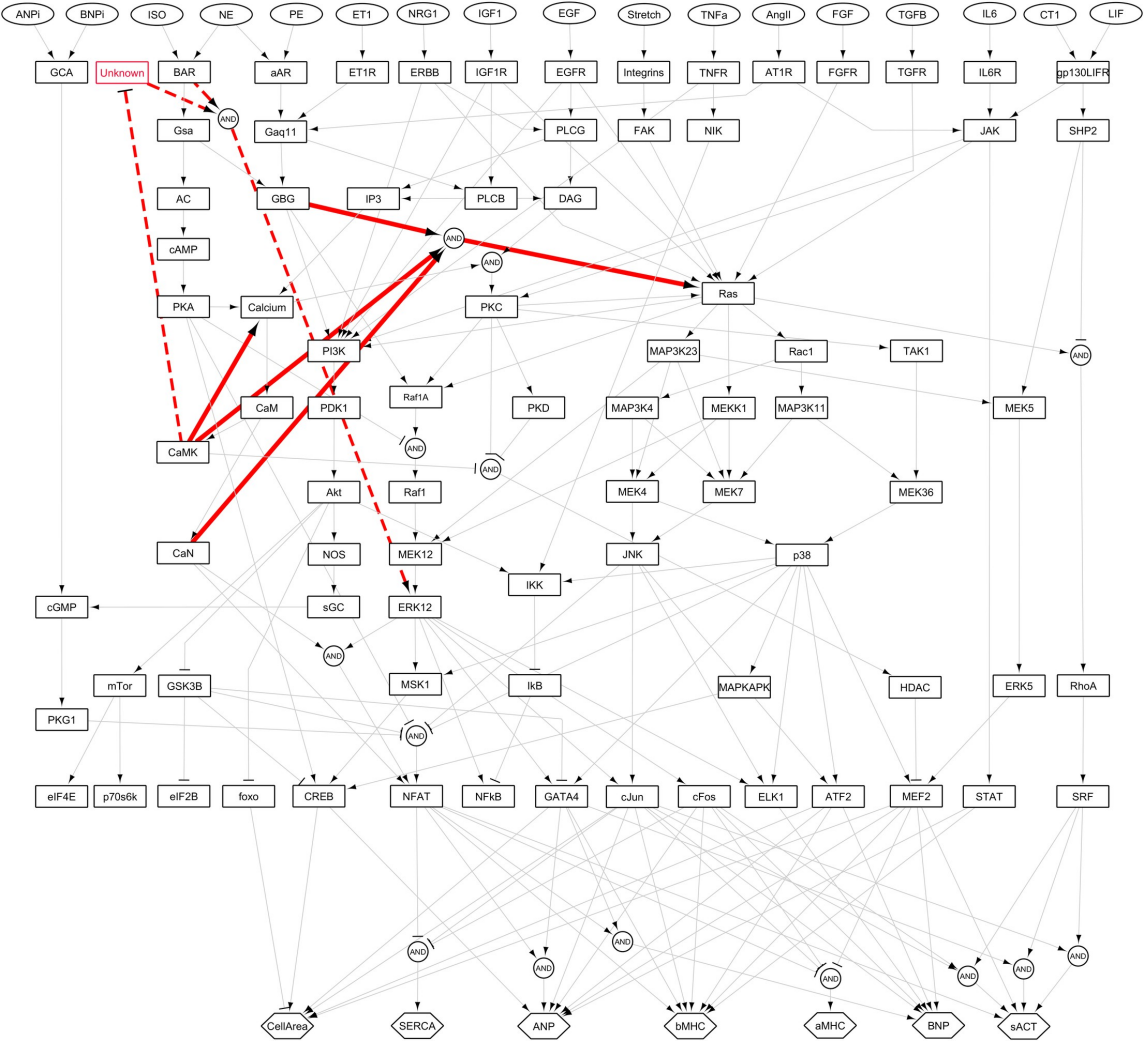
Revised model of β-adrenergic cardiac hypertrophy. The solid red arrows exhibit predicted and validated interactions in ISO-specific context. The dashed red arrows illustrate proposed interactions predicted by model based on the new experimental data on the effects of CaMKII inhibition on ISO-induced ERK1/2 activity.

Based on the experimentally validated inferred interactions, we updated the hypertrophy model for the ISO-specific context (S4 Table) as illustrated in Fig 8. The red solid lines describe the CLASSED-inferred reactions that were experimentally validated in the ISO-specific context. While revision on model parameters enhanced model prediction accuracy from 48% (original model) to 72% in ISO-specific context, adding new interactions increases this prediction accuracy to 86.7% (65 of 75 predictions). Moreover, red dashed-lines in Fig 8 suggest additional new interactions that are consistent with the unanticipated effect of CaMKII inhibition on enhancing ISO-induced ERK1/2 phosphorylation. Consistent with the new suggested interactions in dashed lines, some studies with other cell types have shown that KN93 increases the MEK12/ERK1/2 activity through EGFR activation [65], but this activation is not through Ras/Raf1 [66] and directly influences ERK1/2 activity. Also, there are several studies which confirm the transactivation of other pathways like EGFR during β-adrenergic receptor activation and their Ras-independent influence on ERK1/2 activity [67].

## Discussion

The present study provides a systematic approach, CLASSED, for developing context-specific models for large-scale signaling networks. CLASSED has been designed to systematically revise a prior-knowledge network in accordance with context-specific perturbation data in qualitative or semi-quantitative formats. Given that the logic-based ODE formulation implemented in this approach could capture the dynamics of signaling networks, our method has the potential to accommodate quantitative data. The automated validation module generates LDE models and outputs model prediction accuracy and validation data for each and overall experimental conditions. In this regard, we employed expanded context-specific hypertrophic data curated from the literature and a large-scale hypertrophy network [4,42] to identify the key signaling reactions for model validity in four hypertrophic contexts. We also developed an ISO-specific hypertrophy model through the four-stage model revision process. The ISO-specific model predicted new crosstalks between the calcium/calmodulin-dependent pathways (CaMKII and CaN) and the upstream reactions of Ras, and a positive feedback from CaMKII to calcium. These new interactions were examined and validated by previous experimental data as well as new experiments with primary cardiomyocytes. Our experiments revealed a synergic effect of CaMKII-Gβγ and CaN-Gβγ pairs in regulating ISO-induced ERK1/2 phosphorylation which is an important player of cardiac hypertrophy [68,69].

### Context-specific network model

Context-dependency is a central but poorly understood aspect of cell signaling [70]. Recently, this concept has been emphasized in developing new drug treatments, particularly personalized treatments for cancer patients. In several studies by the Saez-Rodriguez group, the authors showed that patient- or context-specific logic-based ODE models enhanced prediction of cancer cells’ response in comparison with non-context-specific models [40,71,72]. The Alberts group have highlighted the efficacy of discrete dynamic models to understand biological signaling and regulatory networks in different contexts [37]. In this regard, they developed different models to study T cell large granular lymphocyte (T-LGL) survival signaling [73,74], immune response to B. bronchiseptica during its infection of the lower respiratory system [75] and plant guard cell signaling [76] and demonstrated the model efficacy in different biological systems.

Apart from enabling dynamic predictions on large-scale signaling networks, logic-based ODE models have the capability to model context-dependent networks despite limited availability of patient- or context-specific data. Although in some fields data is more plentiful, such as with ex vivo high-throughput screening of cancer biopsies [40] or by widespread data sharing as in the DREAM challenges [77], most fields face challenge in insufficient data where prior-knowledge based models may be helpful. Furthermore, these approaches provide the opportunity to integrate the data from different labs and experimental studies to validate a model.

In our study, the prior-knowledge model reactions are supported with at least two independent studies [4]. For context-specific data, the majority of the experimental data are supported by at least two independent studies. Since diversity of data is very important for model prediction, in parts of the network that limited data are available, using one study that includes data from at least three replicates is acceptable as long as is not in complete disagreement with the other data related to that part of network.

### CaMKII-Gβγ and CaN-Gβγ: new crosstalks in the ISO-specific context

Through systematic revision of the hypertrophy network model, we predicted a new synergistic cross-talk between CaMKII and Gβγ in ISO-induced hypertrophy. This prediction motivated new experiments, which found that while ISO-induced ERK1/2 phosphorylation was not suppressed by pharmacologic inhibition of CaMKII or Gβγ alone, combined inhibition significantly suppresses the ISO-induced ERK1/2 phosphorylation in both cardiac myocytes and fibroblasts. We also experimentally validated similar crosstalk between CaN and Gβγ, which had a more subtle quantitative influence on ERK1/2 phosphorylation. Moreover, while Zou et al. [63] reported no effect of CaMKII on ERK1/2 activity in ISO-specific context, our observations from western blot and immunofluorescence experiments showed an increase in the ERK1/2 activity after CaMKII inhibition with KN93 in both cardiomyocytes and fibroblasts.

The context-specificity of these interactions is emphasized by comparison of our results and those of Cipolletta et al. [62] on CaMKII/ERK1/2 interaction in PE-treated H9C2 cardiomyoblasts. While they found that CaMKII inhibition reduces PE-induced ERK1/2 activity and its nuclear accumulation, our data with ISO-induced ERK1/2 phosphorylation show enhancement by CaMKII inhibition alone or suppression with combined inhibition of CaMKII and Gβγ. In terms of cell type, while a number of studies found similarities in response for neonatal rat cardiomyocytes and H9C2 cardiomyoblasts [78], others have reported distinct responses for these two cell types [79]. In terms of stimuli, the model predicts more contribution of calcium to Ras/Raf1/MEK/ERK1/2 pathway through PKC in PE-specific context than ISO-specific context (S7 Fig). The effect of CaMKII inhibition on calcium could result in the suppression of ERK1/2 activity in PE-specific context. In another study by Illario et al. [80], the authors reported decrease in ERK1/2 phosphorylation after CaMKII inhibition for two non-myocyte cell lines after integrin stimulation by fibronectin. These studies indicate the context-specificity of ERK1/2 response.

In contrast to the few studies on crosstalks between CaMKII and ERK1/2, many studies have explored crosstalks between CaN and ERK1/2. For instance, expression of activated calcineurin transgene in mice showed ERK1/2 activation in the heart [81]. The results of Zou et al. study [63] also showed activation of ERK1/2 signaling through a calcineurin-dependent mechanism in ISO-specific context. On the other hand, several studies reported ERK1/2 signaling can regulate calcineurin–NFAT activity through stimulation or inhibition based on the context and present NFAT factors [60]. In our study, the ISO-specific model predicts the potential effect of CaN on ERK1/2 activity, and the validation experiment indicates the additional requirement of crosstalk with Gβγ.

Furthermore, our experiments showed a significant difference in ISO-induced ERK1/2 phosphorylation between cardiomyocytes and fibroblasts in terms of magnitude and localization (S10 Fig). While in this case both cardiomyocytes and fibroblasts had similar qualitative responses to inhibitors, improving cell purity or utilizing alternate methods like immunofluorescence may be critical in future studies of cell-specific responses.

### Limitations and future directions

While in this study we initially used default parameters for major of signaling reactions, tuning of more reaction parameters can improve the model prediction accuracy as more context-specific data becomes available. The availability of high-quality databases for context-specific data like the current databases in cancer systems biology [82–84] could result in more accurate and robust model predictions. As richness and diversity of data affect model accuracy and its ability to confer new crosstalks, we tried to obtain diverse data from different pathways and various contexts as much as we could. Moreover, adding only a single reaction at a time in the final stage of CLASSED approach limits its capacity to infer new pair interactions with synergic effect in the signaling network.

Although applying the CLASSED approach to the hypertrophy signaling network in the β-adrenergic context resulted in predictions of new crosstalks that were validated by experiments, it is possible that in some cases, experiments do not support model predictions. This inconsistency can be a result of the following limitations: 1- The prediction power of the current approach is dependent on the accuracy of the prior knowledge network and, every false reaction could affect model predictions. 2- Diversity and accuracy of context-specific experimental data considerably affect model predictions. In parts of the network that we do not have enough context-specific data, model predictions should be considered with more caution. 3- Since this approach nominates considerable numbers of reactions as missing interactions, selecting the final cases for validation experiments is not always straightforward and requires comprehensive knowledge of the studied subject.

In this study, we employed our approach to develop an ISO-specific model of cardiac hypertrophy. For the future, curation of more context-specific and semi-quantitative data could enlighten the context-dependent characteristics of cardiac myocytes. Furthermore, validating the predictions of context-specific models with in vivo data could illustrate the contribution of other factors in the specificity of cell response in each context.

## Supporting information

S1 Table Prior-knowledge network model of cardiac hypertrophy. (XLSX)

S2 Table Expanded context-specific qualitative data curated from literature. (XLSX)

S3 Table Semi-quantitative ISO-specific data. (XLSX)

S4 Table Revised network model of β-adrenergic cardiac hypertrophy. (XLSX)

S1 Fig Prior-knowledge cardiac hypertrophy signaling network. The network comprises 106 nodes, 191 reactions and 17 receptor inputs (green color) [4]. Seven nodes have been defined as model outputs (blue color).
(TIF)

S2 Fig Modifications of normalized-Hill LDE approach. (A, B) Comparison of the original [39] and modified versions of the inhibition and (C, D) “AND” gate formula. (E) The sensitivity of model validation percent to *in silico* threshold for determination of a change in model outputs.
(TIF)

S3 Fig Classified qualitative validation of the model. All qualitative data in four classes (A) input-output (B) input-intermediate (C) intermediate overexpression, and (D) intermediate inhibition are compared with model predictions except for ISO-specific context. The red, blue, and gray boxes illustrate increase, decrease, and no change, respectively. In the model, variations of the measured node activity greater than +1% or smaller than -1% have been considered as an increase or decrease, respectively. Statistically significant changes in comparison with control have been considered for variations in experimental data.
(PDF)

S4 Fig Key reactions identified by a single-reaction deletion technique. (A) PE-specific and (B) Stretch-specific contexts have been visualized on the hypertrophy network map. The thicker and bolder red or blue arrows illustrate more decrease or increase in validation percent after removing each reaction, respectively. (C) Categorizing hypertrophy network reactions in three categories of “non-sensitive”, “direct”, and “shared” reactions in ISO, AngII, Stretch and PE-specific contexts.
(TIF)

S5 Fig Changing variant parameter from WR to EC50 and its effects on Morris sensitivity analysis. The left diagram illustrates the Morris index (μ*) and its standard deviation (σ) for each reaction in ISO-specific network by variating the WR parameter. Consequently, important reactions of ISO-specific network are illustrated in the right diagram. Greater Morris index (μ*) illustrates more influence on model validation percent. Larger σ to μ* ratio for each reaction (above μ* = σ diagonal line) demonstrates a more nonlinear effect on model prediction accuracy (interaction with other reactions). The orange dots and blue stars illustrate the reactions with monotonic and non-monotonic effects in ISO-specific network, respectively. The vertical red line shows the significance level for identifying non-important reactions. In the network view, the thicker red arrows illustrate reactions with larger Morris index (μ*).
(TIF)

S6 Fig Morris sensitivity analysis identifies non-important reactions in each context. (A) The Morris index (μ*) and standard deviation (σ) for each reaction (left) and the main reactions for model validity (right) are illustrated for AngII-specific, (B) PE-specific and (C) Stretch-specific contexts. Greater Morris index (μ*) illustrates more influence on model validation percent. Larger σ to μ* ratio for each reaction (above μ* = σ diagonal line) demonstrates a more nonlinear effect on model prediction accuracy (interaction with other reactions). The orange dots and blue stars illustrate the reactions with monotonic and non-monotonic effects in ISO-specific network, respectively. The vertical red line shows the significance level for identifying non- important reactions.
(TIF)

S7 Fig Key reactions identified by Sobol sensitivity analysis in four hypertrophic contexts. (A) The main reactions regulating model validity in ISO-, (B) PE-, (C) AngII—and (D) Stretch-specific contexts are illustrated on the hypertrophy network map. Thicker red arrows illustrate the larger Total Sobol sensitivity index.
(TIF)

S8 Fig Validation of ISO-specific network model with qualitative and semi-qualitative data. All data in ISO-specific context (75 qualitative and 100 semi-qualitative) are compared with model predictions before and after tuning of 12 reaction parameters (EC50). Semi-qualitative data has been categorized in 3 levels of increase (LH: Low High (1.01–2 folds), MH: Medium High (2–5 folds), HH: High High (higher than 5 folds)) and decrease (LL: Low Low (0.5–0.99 folds), ML: Medium Low (0.2–0.5 folds), HL: High Low (less than 0.2 folds)). See S3 Table for detailed information.
(PDF)

S9 Fig Nominations of Calcineurin crosstalks in ISO-specific network. (A) Crosstalks identified by model between CaN and hypertrophy network reactions with the “AND” gate before and (B) after adding positive feedback predicted by model from CaMKII to Calcium. The thicker red arrows illustrate reactions with higher positive effect on ISO-specific model validation percent.
(TIF)

S10 Fig Discrepancy in ISO-induced ERK1/2 response for cardiac myocytes and fibroblasts. (A) The immunofluorescence results display different ERK1/2 phosphorylation in ISO-specific context in terms of translocation and (B) magnitude between cardiac myocytes and fibroblasts. The single and double hashtags (#) display statistically significant changes (p<0.05) after ISO stimulation for cardiac fibroblasts and myocytes, respectively, in comparison with control (DMSO). The single and double stars (*) exhibit statistically significant changes (p<0.05) for cardiac fibroblasts and myocytes, respectively, in comparison with ISO. Data were collected from 3 independent experiments (Mean±SEM)
(TIF)
