# Supplementary figures and images for: Context-specific network modeling identifies new crosstalk in β-adrenergic cardiac hypertrophy
Source: PLoS Comput Biol. 2020 Dec 18;16(12):e1008490. doi: 10.1371/journal.pcbi.1008490 (PMC7781532; doi:10.1371/journal.pcbi.1008490)

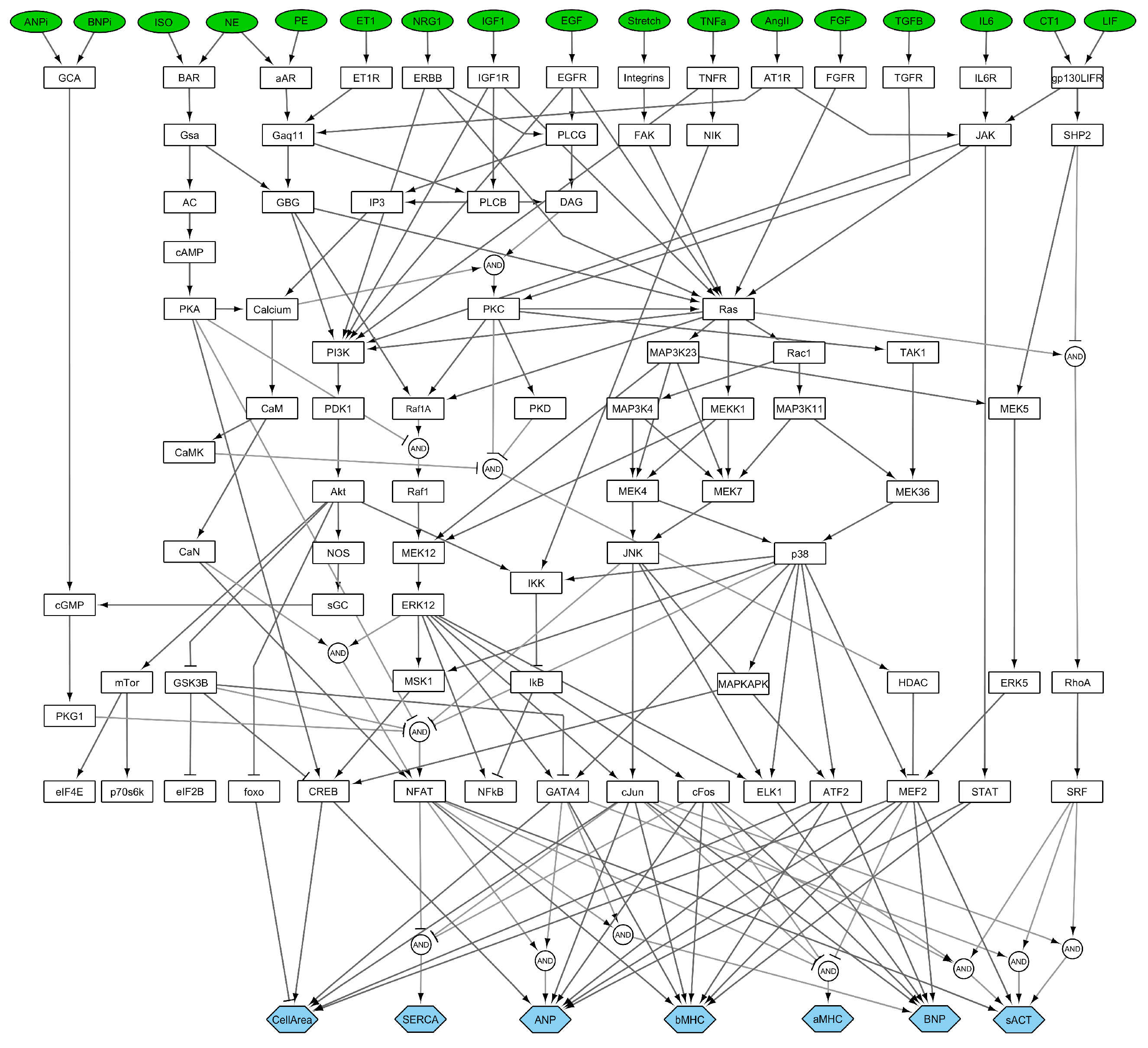

Supplement: S1 Fig — The network comprises 106 nodes, 191 reactions and 17 receptor inputs (green color) [4]. Seven nodes have been defined as model outputs (blue color). (TIF) [file pcbi.1008490.s005.tif]

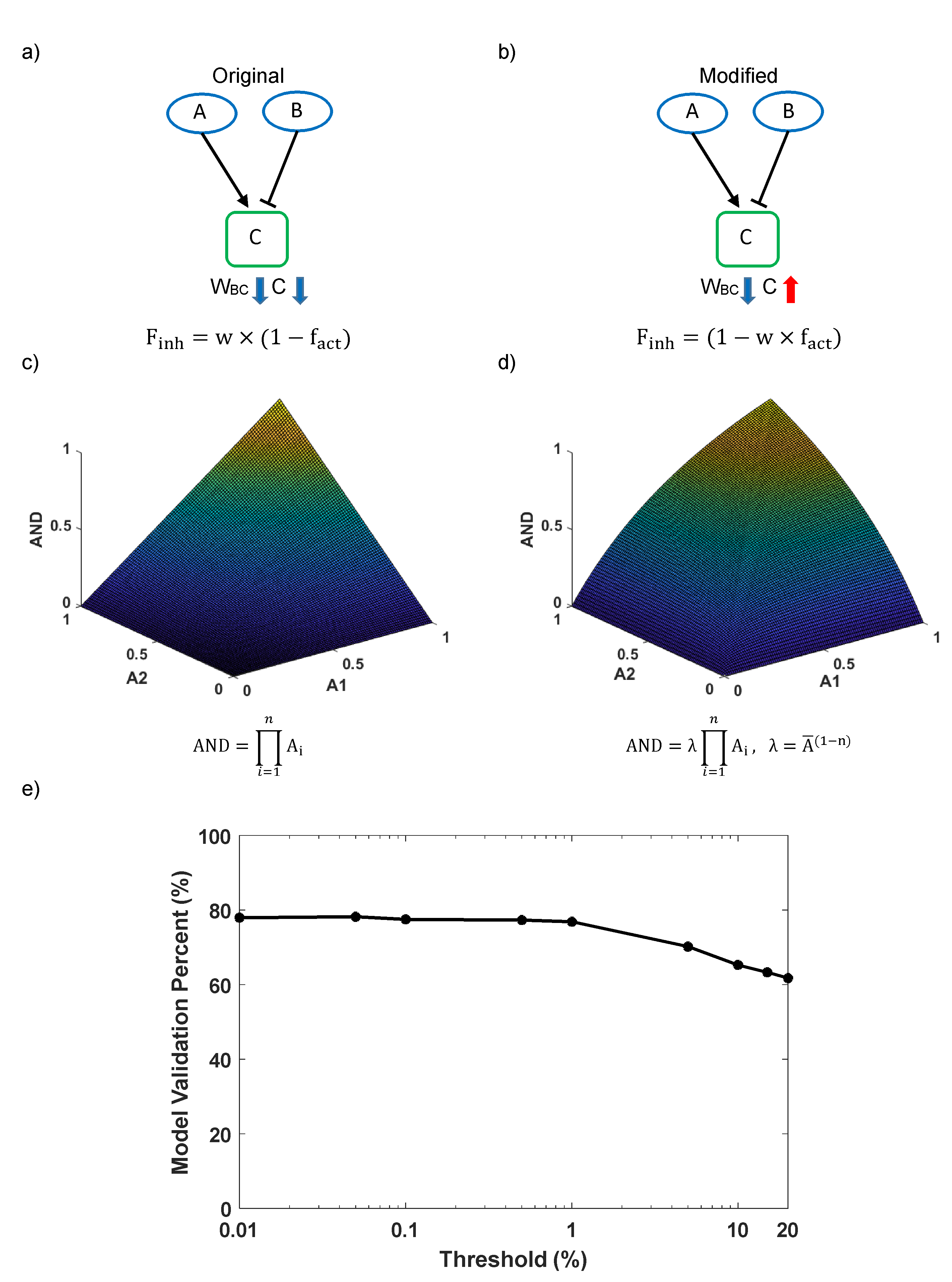

Supplement: S2 Fig — (A, B) Comparison of the original [39] and modified versions of the inhibition and (C, D) “AND” gate formula. (E) The sensitivity of model validation percent to in silico threshold for determination of a change in model outputs. (TIF) [file pcbi.1008490.s006.tif]

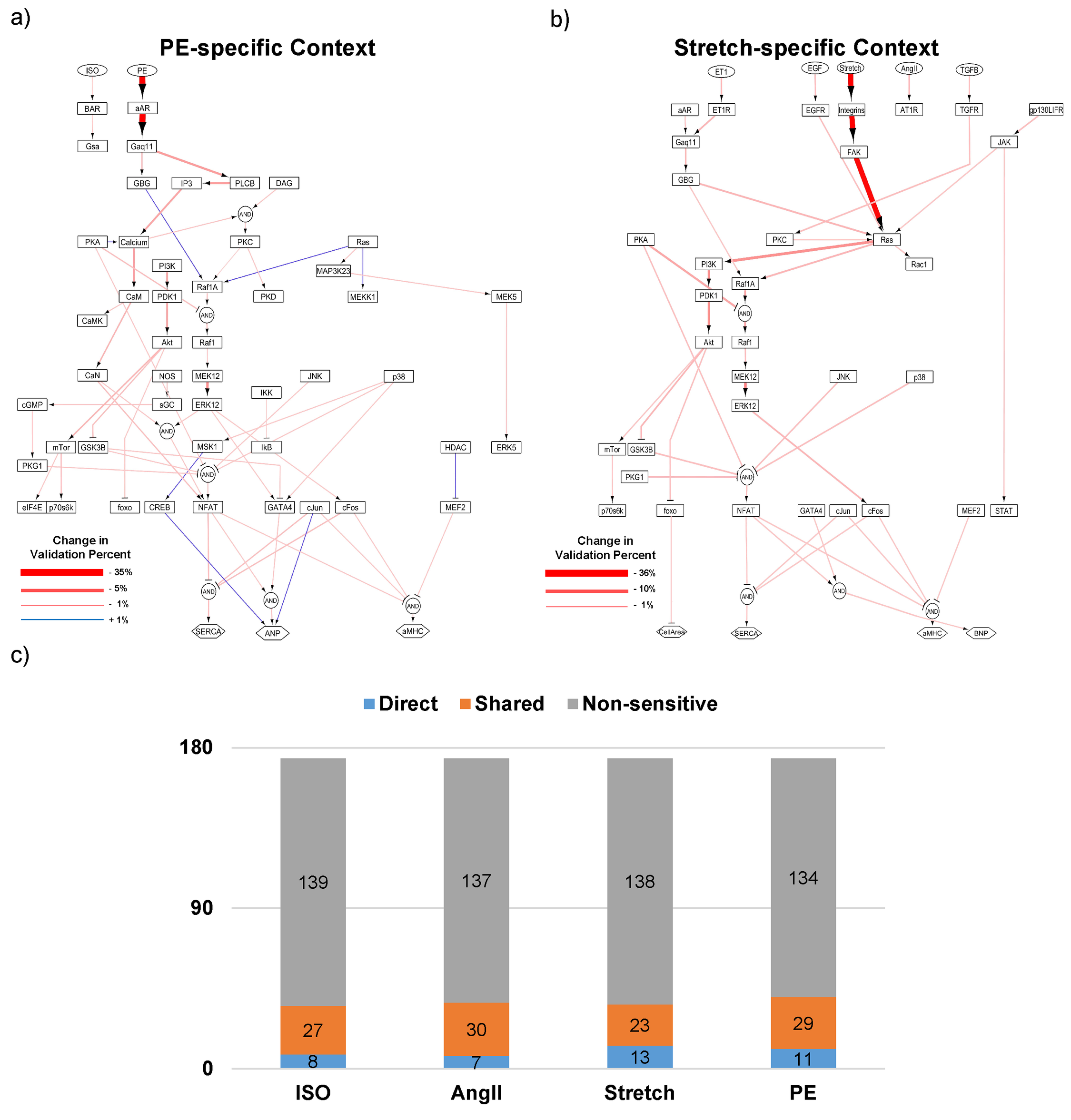

Supplement: S4 Fig — (A) PE-specific and (B) Stretch-specific contexts have been visualized on the hypertrophy network map. The thicker and bolder red or blue arrows illustrate more decrease or increase in validation percent after removing each reaction, respectively. (C) Categorizing hypertrophy network reactions in three categories of “non-sensitive”, “direct”, and “shared” reactions in ISO, AngII, Stretch and PE-specific contexts. (TIF) [file pcbi.1008490.s008.tif]

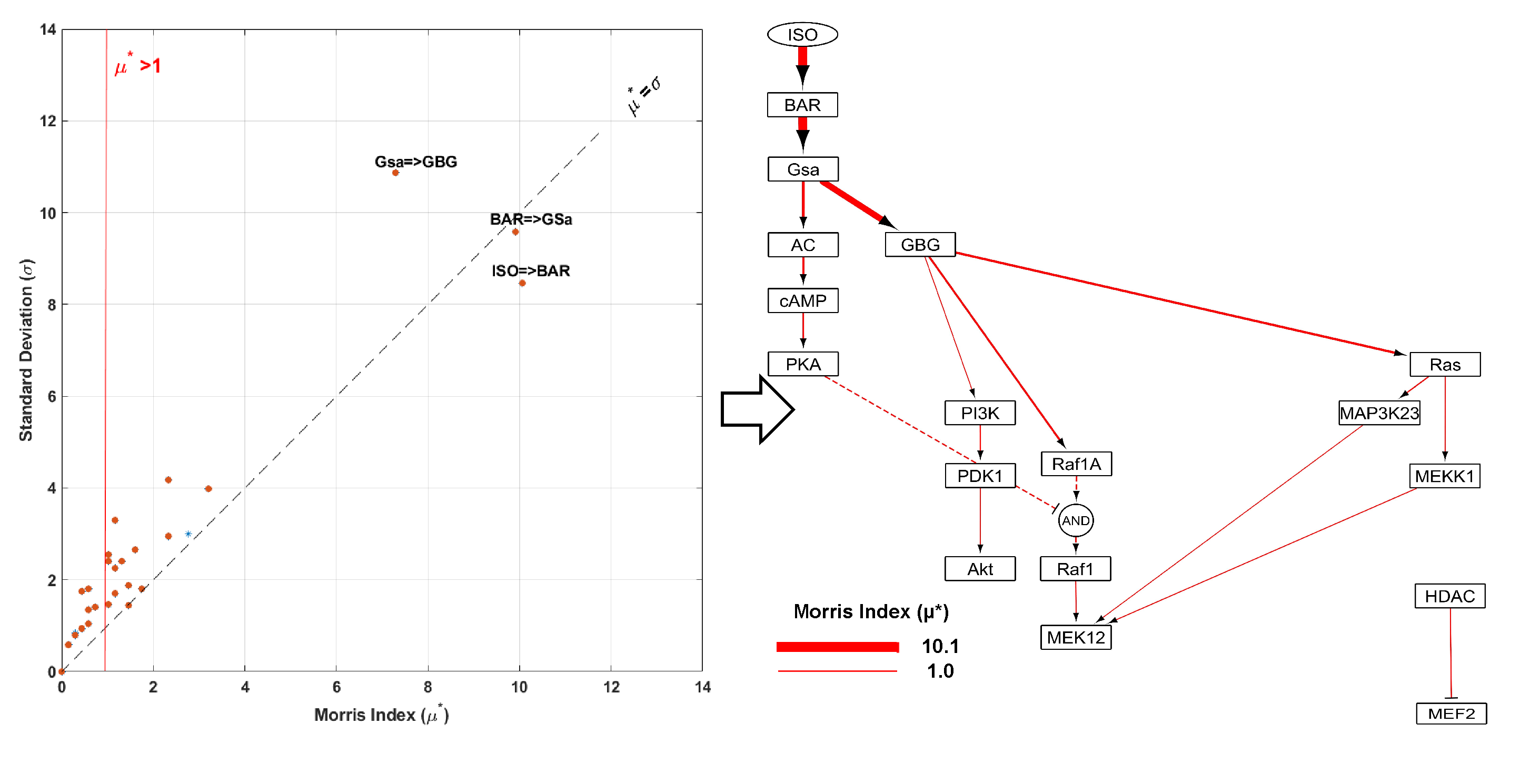

Supplement: S5 Fig — The left diagram illustrates the Morris index (μ*) and its standard deviation (σ) for each reaction in ISO-specific network by variating the WR parameter. Consequently, important reactions of ISO-specific network are illustrated in the right diagram. Greater Morris index (μ*) illustrates more influence on model validation percent. Larger σ to μ* ratio for each reaction (above μ* = σ diagonal line) demonstrates a more nonlinear effect on model prediction accuracy (interaction with other reactions). The orange dots and blue stars illustrate the reactions with monotonic and non-monotonic effects in ISO-specific network, respectively. The vertical red line shows the significance level for identifying non-important reactions. In the network view, the thicker red arrows illustrate reactions with larger Morris index (μ*). (TIF) [file pcbi.1008490.s009.tif]

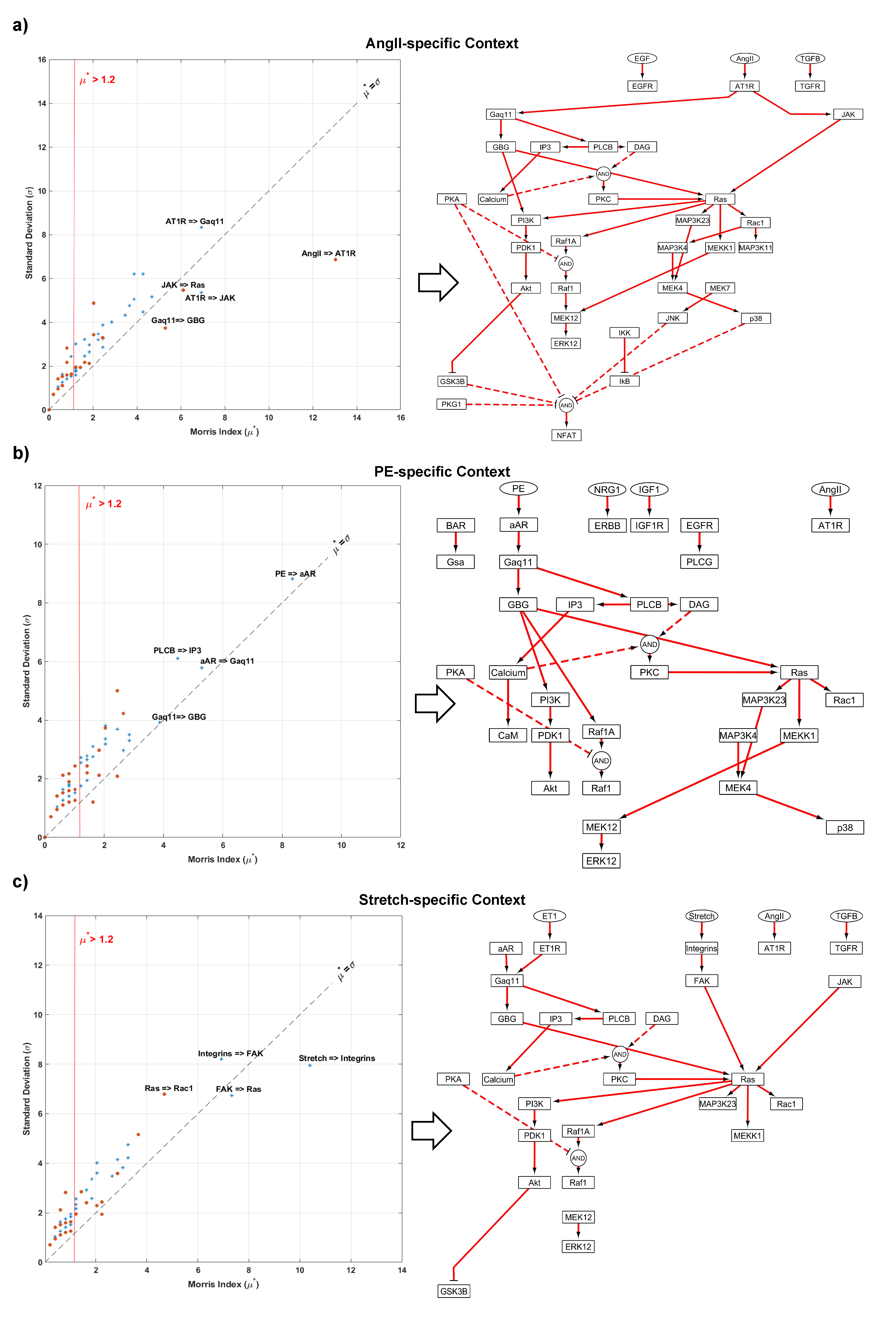

Supplement: S6 Fig — (A) The Morris index (μ*) and standard deviation (σ) for each reaction (left) and the main reactions for model validity (right) are illustrated for AngII-specific, (B) PE-specific and (C) Stretch-specific contexts. Greater Morris index (μ*) illustrates more influence on model validation percent. Larger σ to μ* ratio for each reaction (above μ* = σ diagonal line) demonstrates a more nonlinear effect on model prediction accuracy (interaction with other reactions). The orange dots and blue stars illustrate the reactions with monotonic and non-monotonic effects in ISO-specific network, respectively. The vertical red line shows the significance level for identifying non- important reactions. (TIF) [file pcbi.1008490.s010.tif]

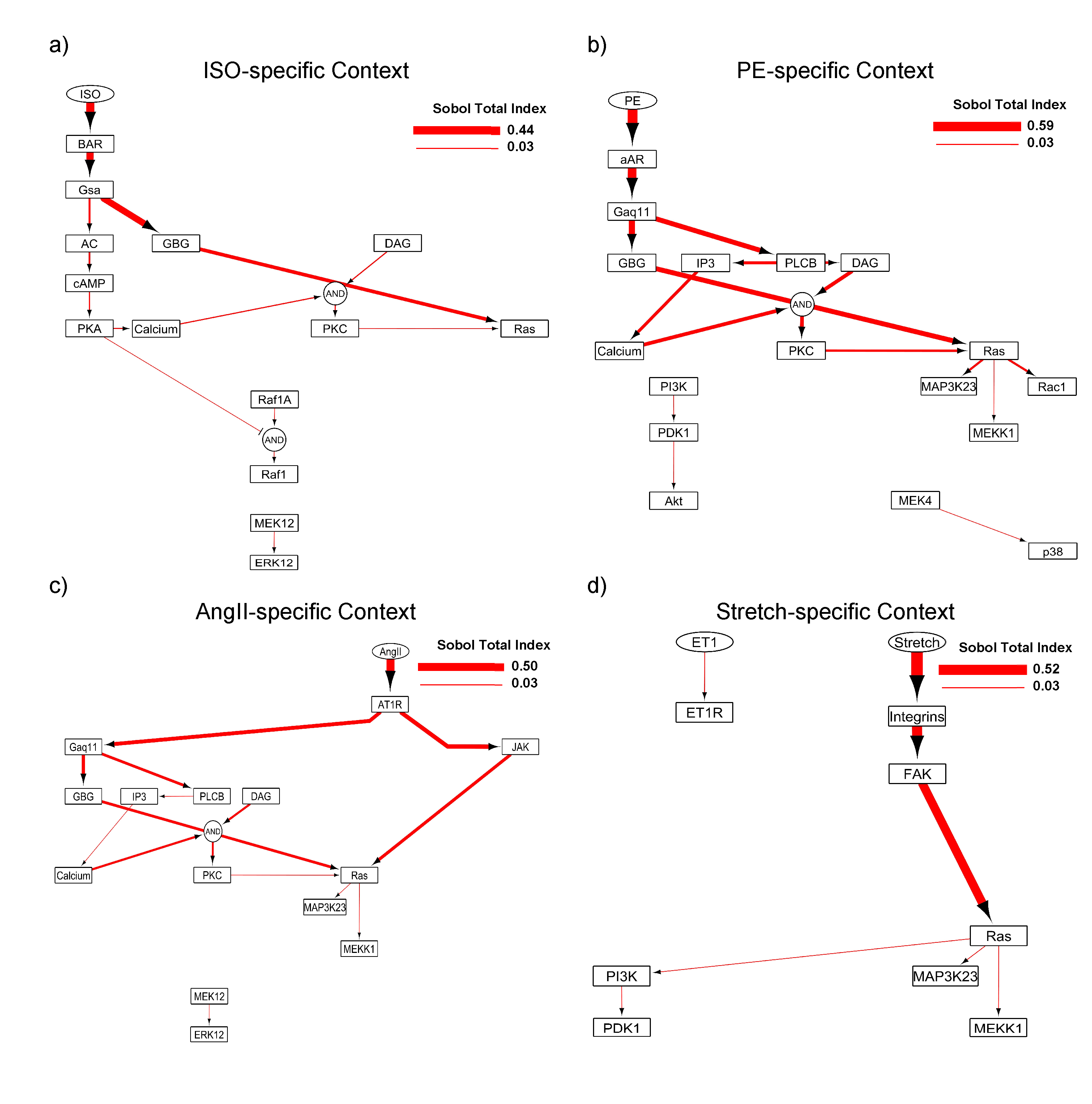

Supplement: S7 Fig — (A) The main reactions regulating model validity in ISO-, (B) PE-, (C) AngII—and (D) Stretch-specific contexts are illustrated on the hypertrophy network map. Thicker red arrows illustrate the larger Total Sobol sensitivity index. (TIF) [file pcbi.1008490.s011.tif]

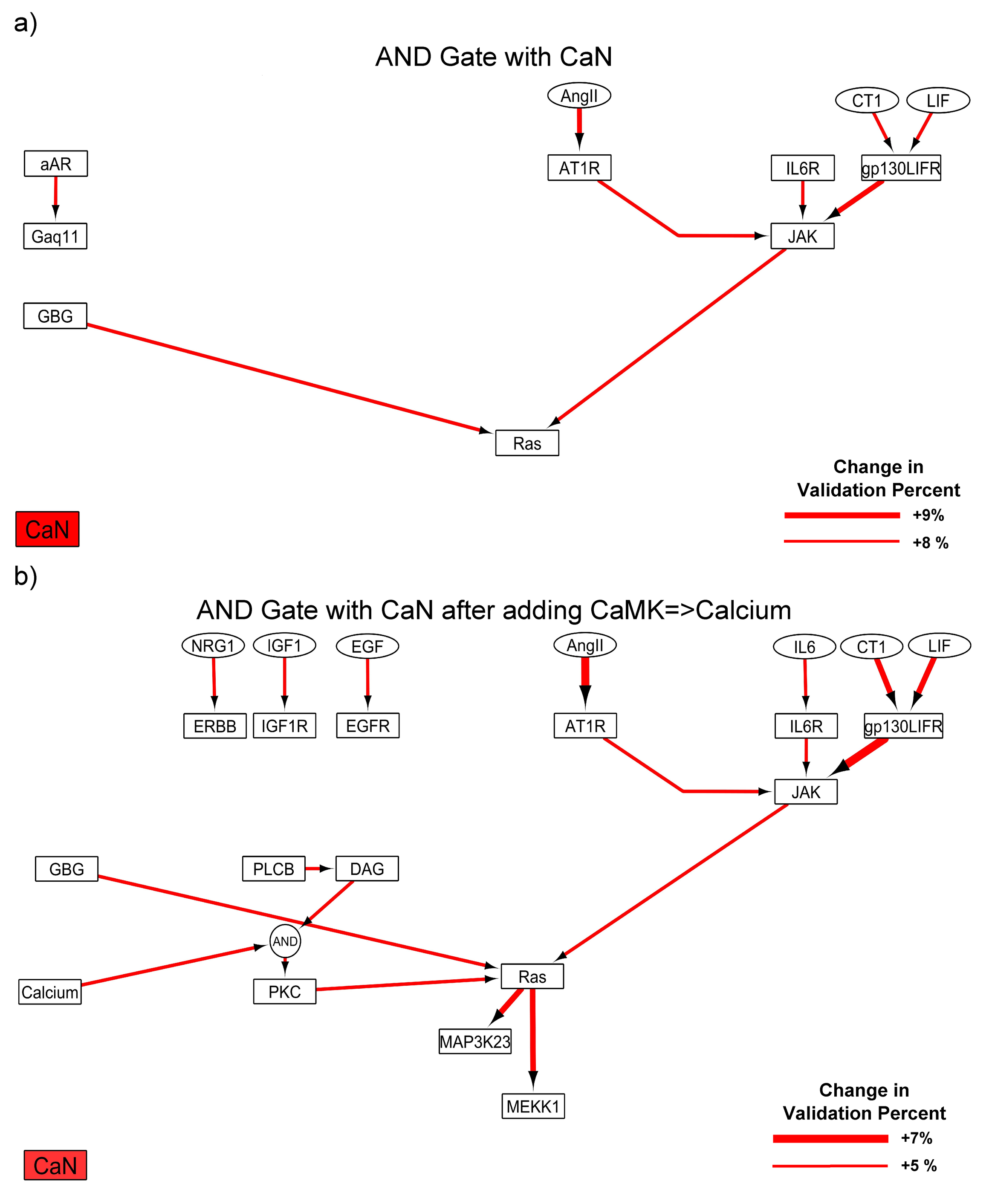

Supplement: S9 Fig — (A) Crosstalks identified by model between CaN and hypertrophy network reactions with the “AND” gate before and (B) after adding positive feedback predicted by model from CaMKII to Calcium. The thicker red arrows illustrate reactions with higher positive effect on ISO-specific model validation percent. (TIF) [file pcbi.1008490.s013.tif]

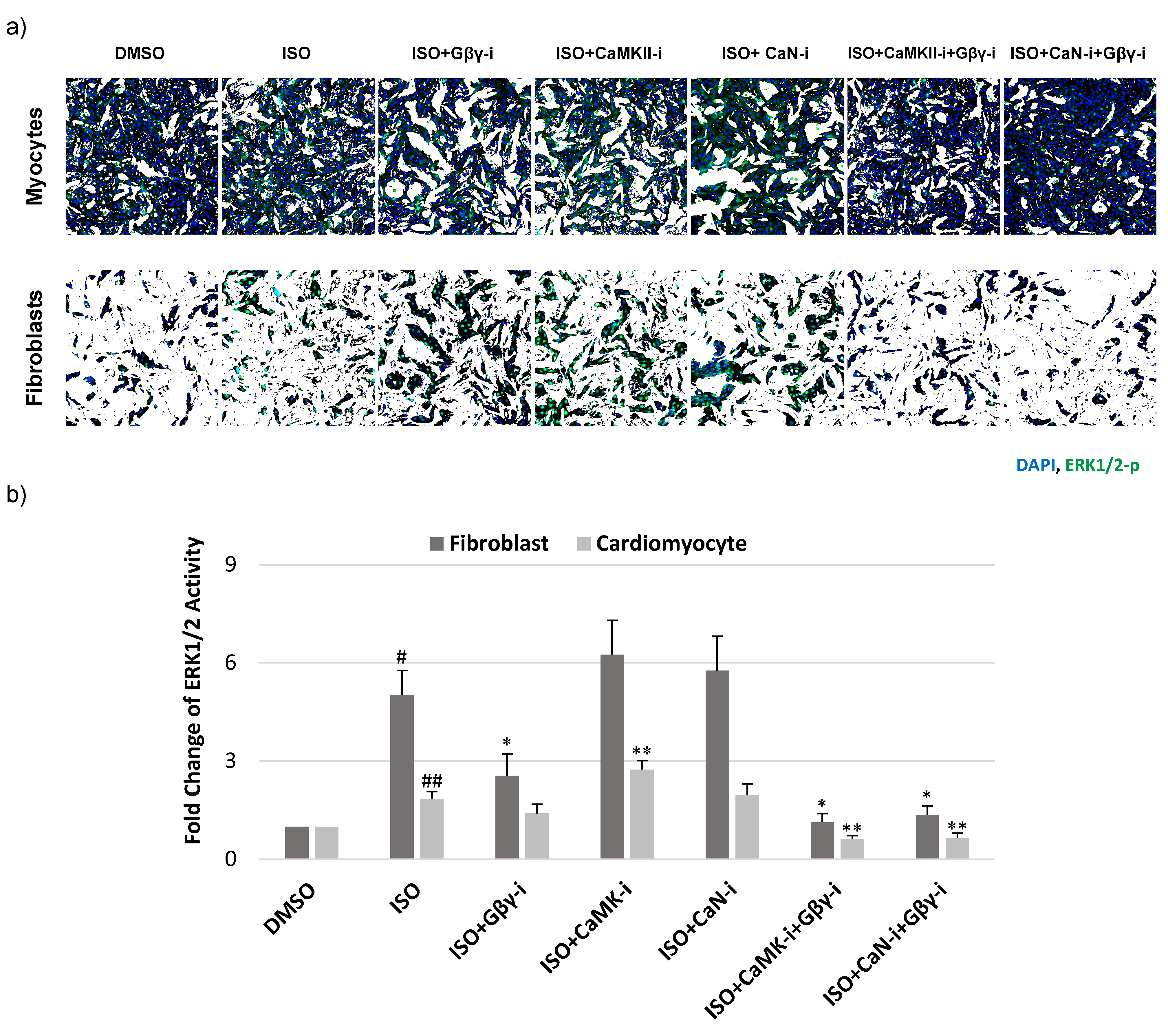

Supplement: S10 Fig — (A) The immunofluorescence results display different ERK1/2 phosphorylation in ISO-specific context in terms of translocation and (B) magnitude between cardiac myocytes and fibroblasts. The single and double hashtags (#) display statistically significant changes (p<0.05) after ISO stimulation for cardiac fibroblasts and myocytes, respectively, in comparison with control (DMSO). The single and double stars (*) exhibit statistically significant changes (p<0.05) for cardiac fibroblasts and myocytes, respectively, in comparison with ISO. Data were collected from 3 independent experiments (Mean±SEM) (TIF) [file pcbi.1008490.s014.tif]
