# Supplementary material for: Context-specific network modeling identifies new crosstalk in β-adrenergic cardiac hypertrophy
Source: PLoS Comput Biol. 2020 Dec 18;16(12):e1008490. doi: 10.1371/journal.pcbi.1008490 (PMC7781532; doi:10.1371/journal.pcbi.1008490)
Supplement: S3 Fig — All qualitative data in four classes (A) input-output (B) input-intermediate (C) intermediate overexpression, and (D) intermediate inhibition are compared with model predictions except for ISO-specific context. The red, blue, and gray boxes illustrate increase, decrease, and no change, respectively. In the model, variations of the measured node activity greater than +1% or smaller than -1% have been considered as an increase or decrease, respectively. Statistically significant changes in comparison with control have been considered for variations in experimental data. (PDF) [file pcbi.1008490.s007.pdf]

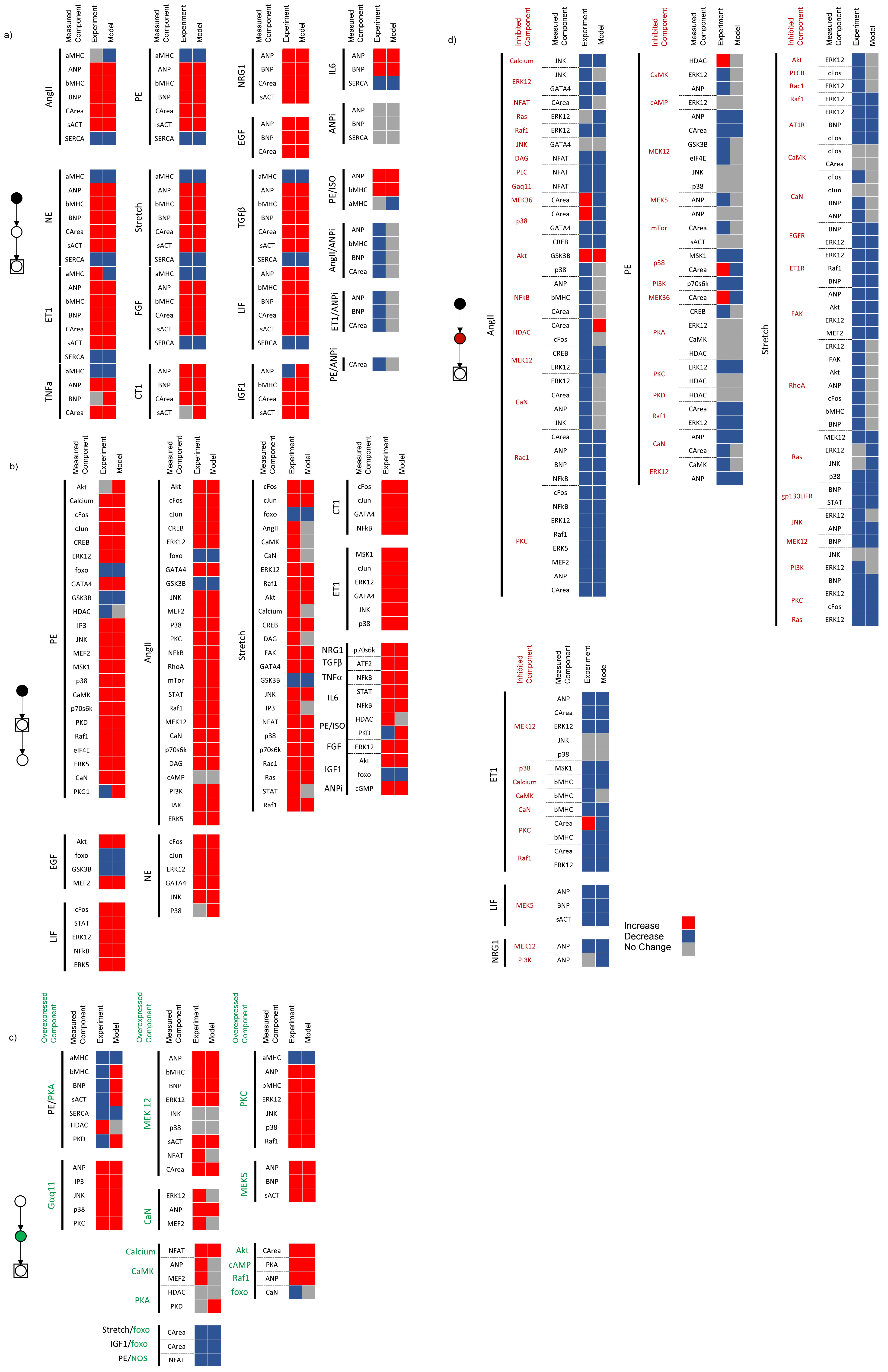

a)
Measured Component
Experiment
Model
AngII
PE
NRG1
EGF
IL6
ANPi
NE
Stretch
TGFβ
PE/ISO
AngII/ANPi
ET1
FGF
LIF
ET1/ANPi
PE/ANPi
TNFa
CT1
IGF1
b)
PE
AngII
Stretch
CT1
ET1
NRG1
TGFβ
TNFα
IL6
PE/ISO
FGF
IGF1
ANPi
EGF
NE
LIF
c)
Overexpressed Component
PE/PKA
MEK 12
PKC
Gαq11
CaN
MEK5
Calcium
CaMK
PKA
Akt
cAMP
Raf1
foxo
Stretch/foxo
IGF1/foxo
PE/NOS
d)
Inhibited Component
AngII
PE
Stretch
ET1
LIF
NRG1
Increase
Decrease
No Change
