# Supplementary material for: Context-specific network modeling identifies new crosstalk in β-adrenergic cardiac hypertrophy
Source: PLoS Comput Biol. 2020 Dec 18;16(12):e1008490. doi: 10.1371/journal.pcbi.1008490 (PMC7781532; doi:10.1371/journal.pcbi.1008490)
Supplement: S8 Fig — All data in ISO-specific context (75 qualitative and 100 semi-qualitative) are compared with model predictions before and after tuning of 12 reaction parameters (EC50). Semi-qualitative data has been categorized in 3 levels of increase (LH: Low High (1.01–2 folds), MH: Medium High (2–5 folds), HH: High High (higher than 5 folds)) and decrease (LL: Low Low (0.5–0.99 folds), ML: Medium Low (0.2–0.5 folds), HL: High Low (less than 0.2 folds)). See S3 Table for detailed information. (PDF) [file pcbi.1008490.s012.pdf]

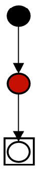

ISO

Inhibited Component	Measured Component	Experiment	Not-tuned Model	Tuned Model
aAR	aMHC		LH	LH
	ANP			
	bMHC			
Akt	ANP	ML		
AT1R	CREB			LL
	ERK12	ML		LL
	JNK	LL		LL
	MEK12	ML		LL
	p38	LL		LL
	Raf1	ML		LL
Calcium	ANP			
	BNP			
	ERK12			
	sACT			
CaMK	Akt			
	aMHC			
	ANP			
	ANP	ML		
	bMHC			
	BNP			
	Calcium			
	Calcium	LL		
	CArea			
	ERK12			
	sACT			
cAMP	ERK12			
	ERK12	ML		LL
CaN	NFAT			
	CArea			
	ERK12			
	Raf1			
	ANP	LL		
	Calcium	LL		
	CArea	LL		
	ERK12	ML		

ISO

Inhibited Component	Measured Component	Experiment	Not-tuned Model	Tuned Model
GBG	ERK12			
HDAC	CArea			
MEK12	CREB			
AC	cAMP			
NFkB	CArea			
Gaq11	ERK12			LL
CaM	ERK12			
p38	ANP			
PI3K	Akt			
	Akt	HL	HL	HL
	Akt	ML	HL	HL
	ANP			
	ANP	HL		
	ERK12			
PKA	aMHC			
	aMHC		HL	LH
	bMHC			
	BNP			
	ERK12			
	ERK12	ML		LL
	SERCA			
PKC	ERK12			
PKD	CArea			
	CArea	LL		
	NFAT	LL		
Raf1	ERK12			
	ERK12	ML		LL
Ras	ERK12	ML	HL	HL
	NFAT			
ERK12	ANP			
	ANP	ML		
	CArea			
	CArea	LL		
	Calcium			

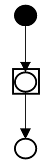

ISO

Measured Component	Experiment	Not-tuned Model	Tuned Model
AC			
AC	MH	HH	LH
AC	HH	HH	HH
AC	HH	HH	HH
AC	HH	HH	HH
AC	HH	HH	HH
AC	HH	HH	HH
Akt			
Akt	MH	HH	MH
Akt	MH	HH	MH
ATF2	LH	HH	LH
Calcium			
Calcium	MH	HH	MH
Calcium	MH	MH	LH
Calcium	MH	HH	MH
Calcium	MH	HH	MH
CaM			
CaMK			
CaMK	LH	HH	HH
cAMP			
cAMP	LH	HH	LH
cAMP	HH	HH	HH
cAMP	HH	HH	HH
cAMP	HH	HH	HH
cAMP	HH	HH	HH
CaN			
CaN	LH	HH	HH
CaN	LH	HH	HH
cFos			
cJun		HH	LH
CREB			
CREB	MH	HH	LH
CREB	MH	HH	LH
CREB	MH	HH	MH
CREB	MH	HH	MH
ELK1			
ELK1	LH	HH	LH
Ras			
Ras	MH	HH	MH

ISO

Measured Component	Experiment	Not-tuned Model	Tuned Model
ERK12			
ERK12	MH	HH	MH
ERK12	MH	HH	MH
ERK12	LH	HH	MH
GATA4			
GATA4	LH	HH	MH
GBG			
GSK3B			
GSK3B	LL	HL	LL
HDAC			
JAK			
JAK	LH		
JNK			
JNK	MH	HH	MH
MEK12			
MEK12	MH	HH	MH
NFAT			
NFAT	LH	HH	LH
NFAT	LH	HH	LH
p38			
p38	MH	HH	MH
p38	LH	HH	MH
p70s6k			
p70s6k	MH	HH	MH
PI3K			
PI3K	MH	HH	MH
PKA			
PKA	HH	HH	HH
PKC			
PKC	LH	LH	LH
PKD			
PKD	LH	LH	LH
Rac1			
Raf1			
Raf1	MH	HL	MH
STAT			
STAT	LH		

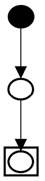

ISO

Measured Component	Experiment	Not-tuned Model	Tuned Model
aMHC			
aMHC	LL		LL
aMHC	LL	HL	LL
aMHC	LL	HL	ML
ANP			
ANP	MH	HH	LH
ANP	LH	HH	LH
ANP	LH	HH	LH
ANP	MH	HH	LH
bMHC			
bMHC	LH	HH	LH
bMHC	LH	HH	LH
bMHC	LH	HH	LH
bMHC	MH	HH	LH
BNP			
BNP	MH	HH	LH
BNP	ML		
CArea			
CArea	LH	HH	LH
CArea	LH	HH	LH
CArea	LH	HH	LH
CArea	LH	HH	LH
sACT			
sACT	LH	HH	LH
SERCA			
SERCA	LL	HL	LL

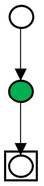

ISO

Overexpressed Component	Measured Component	Experiment	Not-tuned Model	Tuned Model
PE	PKD	MH	HH	[illegible]
PDK1	ANP	MH		
Akt	ANP	LH		
GSK3B	ANP	ML		

Legend	
Increase	
High High	HH
Medium High	MH
Low High	LH
No Change	
Low Low	LL
Medium Low	ML
High Low	HL
Decrease	
